# Mechanically Active Hydrogel for Healing Intestinal Fistulas through the YAP‐Mediated Mechanosensitization of Intestinal Epithelial Cells

**DOI:** 10.1002/advs.202510717

**Published:** 2025-08-11

**Authors:** Ze Li, Jiayang Li, Kang Chen, Guiwen Qu, Shuanghong Yang, Sicheng Li, Ye Liu, Yitian Teng, Rui Ma, Jinjian Huang, Peige Wang, Jianan Ren, Xiuwen Wu

**Affiliations:** ^1^ Research Institute of General Surgery, Jinling Hospital, Affiliated Hospital of Medical School Nanjing University Nanjing 210002 P. R. China; ^2^ Department of Emergency Surgery The Affiliated Hospital of Qingdao University Qingdao 266000 P. R. China; ^3^ Research Institute of General Surgery Jinling HospitalNanjing, Medical University Nanjing 210002 P. R. China; ^4^ School of Medicine Southeast University Nanjing 211189 P. R. China; ^5^ Jinling Clinical Medical College Nanjing University of Chinese Medicine Nanjing 210002 P. R. China

**Keywords:** hydrogel, IEC, intestinal fistula, mechanically active, tissue repair

## Abstract

Intestinal fistula is a serious condition characterized by abnormal connections between the intestine and surrounding tissues. Despite advances in comprehensive management of intestinal fistulas, patients often need to undergo definitive surgery and face life‐threatening complications due to ineffective closure measures. Herein, a mechanically active hydrogel (GNGP) that actively constricts intestinal fistulas in response to body temperature is presented. It possesses broad‐spectrum antimicrobial properties, excellent adhesion, suitable degradation properties, and a balance between injectable properties and mechanical activity—which, together, enable the in situ sealing of intestinal fistulas. Moreover, GNGP hydrogel accelerates the re‐epithelialization process of rabbit intestinal fistulas, ameliorates the inflammation of the fistula tract, and promotes the deposition of collagen into the damaged area to effectively facilitate intestinal fistula healing. Mechanistically, shear stress is found to promote the proliferation of intestinal epithelial cells via Yes‐associated protein‐mediated mechanosensitization, through finite element simulations and in vitro experiments. Together, this mechanically active hydrogel with injectable properties offers a promising strategy for the sealing treatment of intestinal fistulas.

## Graphical abstract

This study develops the hydrogel with antimicrobial, adhesive, degradable, injectable, and temperature‐sensitive contraction properties based on the transcriptomic and pathological characteristics of intestinal fistula patients. This hydrogel can seal rabbit intestinal fistula in situ, reduce the intestinal defect, and promote intestinal epithelial cell proliferation through YAP‐mediated mechanosensitization for intestinal fistula healing.

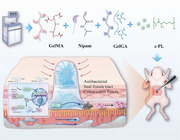

## 1 Introduction

Intestinal fistula is a serious condition characterized by the formation of an abnormal connection between the intestine and the surrounding tissues. This condition is prevalent in missed enterotomies, anastomotic leaks, inflammatory bowel disease, and radiation bowel disease.^[^ ^1^ ^]^ It prevents the implementation of enteral nutrition and predisposes patients to infections, sepsis, and even multiple organ dysfunction syndrome—leading to high mortality rates.^[^ ^2^ ^]^ Although traditional treatments such as abdominal irrigation, negative pressure drainage, nutritional support, and functional exercises may alleviate this condition to some degree, definitive surgical resection of the intestinal fistula is still required in most cases.^[^ ^3^ ^]^ Moreover, intestinal fistula healing remains challenging even with adequate irrigation and drainage, sealing treatments such as fibrin glue, or autologous platelet‐rich plasma transfusion.^[^ ^4^ ^]^ The main obstacles to effective sealing treatments for intestinal fistulas include impaired mucosal repair of the intestinal fistula, fibrosis of the sinus tract, bacterial colonization, and the corrosive nature of intestinal fluid.^[^ ^2^, ^5^ ^]^

The proliferation and migration of intestinal epithelial cells (IECs) play crucial roles in the repair of intestinal damage.^[^ ^6^ ^]^ Bridging IEC gaps is crucial for efficiently repairing intestinal damage. Hydrogels have porous network structures similar to that of the extracellular matrix, and thus hold promising potential as tissue engineering scaffolds for promoting mucosal IEC bridging and tissue repair.^[^ ^7^ ^]^ Several studies have shown that injectable hydrogels have promising clinical applications for sealing intestinal fistulas.^[^ ^8^ ^]^ However, the challenges of degradation, antidigestibility, and stable adhesion interfaces of these hydrogels have limited the clinical use of these hydrogels in intestinal fistula sealing therapy.

Biochemical cues in the cellular microenvironment also play important regulatory roles in tissue repair.^[^ ^9^ ^]^ Recent studies have revealed a close interaction between biophysical and biochemical cues, which together influence tissue regeneration and repair.^[^ ^10^ ^]^ For example, if an expandable polymer scaffold is injected into a subcutaneous area and the surrounding skin is pulled,^[^ ^11^ ^]^ the resultant shear stress can be sensed by cytoskeletal regulators and act upstream of stress‐converting molecules. This, in turn, can lead to non‐destructive skin expansion. In a previous study, we also found that temperature‐sensitive hydrogels could exert shear stress on skin wounds through their own contraction, and that this shear stress could reduce the area of damaged tissues and upregulate the proliferative activity of skin basal cells, thus promoting wound healing.^[^ ^12^ ^]^ Following intestinal injury, the intestinal mucosa tends to become evert, as a result of being pulled on by surrounding muscles.^[^ ^13^ ^]^ This can lead to an increase in the damaged area, and affect tissue repair.

In this study, we developed a mechanically active hydrogel (GNGP) to address the challenges associated with sealing treatments for intestinal fistulas. It uses temperature‐sensitive contractile behavior to shrink defective tissues and intervene in cell fates, in order to influence disease prognosis. The hydrogel was named from its main components: gelatin methacrylate (GelMA), N‐isopropyl acrylamide (Nipam), gelatin gallic acid (GGA), and polylysine (ε‐PL). The antibacterial peptide ε‐PL was introduced to enhance the hydrogel's antimicrobial properties, guided by the RNA expression profiles of intestinal fistula tissues, according to the biologically driven design concept. The synergistic effect of injectability and temperature‐sensitive shrinkage of the hydrogel was achieved by using the temperature‐sensitive phase change properties of Nipam in combination with the stiffness of GelMA and the low‐temperature physical cross‐linking properties of gelatin. The gel's adhesive strength was enhanced through bionic wet‐bonding with GGA. The GelMA introduced into the Nipam network was able to optimize the gel's properties in terms of degradation. GNGP was found to be effective in promoting intestinal fistula repair after the administration of a sealing treatment in a rabbit model of intestinal fistula. Morphological changes to rabbit intestinal mucosa were analyzed through finite element simulation. Finally, shear stress stimulation of IEC was performed in vitro, which was found to promote the self‐proliferation of IECs through Yes‐associated protein (YAP)‐mediated mechanosensitization. These findings suggest that the GNGP hydrogel, as a mechanically active material, holds promising potential for the sealing treatment of fistula conditions, such as in intestinal fistula repair.

## 2 Results and Discussion

### 2.1 Genetic and Molecular Features of Intestinal Fistulas

**Figure 1:**
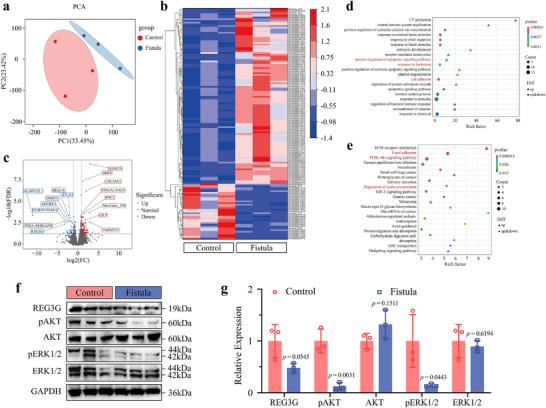
RNA sequencing of clinical intestinal fistula samples. a) Principal component analysis for intestines with fistulas versus normal intestines. b) Heat map of intestinal fistulas versus normal intestine. c) Volcano diagram. The genetic expression of *REG3G*, an antimicrobial peptide involved in intrinsic immunity, was significantly downregulated in the fistula intestines. d) Gene ontology bioprocess analysis. The differentially expressed genes in the fistula intestine were determined to be enriched in the regulation of apoptosis, bacterial response, and cell adhesion compared to the normal intestine. e) KEGG analysis. Differentially expressed genes in the fistula intestine were found to be enriched in focal adhesion, the PI3K‐AKT signaling pathway, and regulation of the actin cytoskeleton compared to the normal intestine. f,g) Western blot and corresponding quantitative analysis of fistula intestines and normal intestines (*n* = 3). The protein expression levels of REG3G, pAKT, and pERK were downregulated in the fistula intestines compared to the normal ones. The *p*‐values in (g) are determined by two‐sided unpaired *t*‐test. Data are presented as mean ± SD.

We performed a comparative analysis of RNA isolated from patient intestinal fistula and normal intestinal tissues via high‐throughput sequencing. Principal component analysis and heat maps of differentially expressed genes (DEGs) demonstrated that the intestinal fistula differed significantly from the normal intestinal tissues in terms of gene expression (**Figure** **1**a,b). Volcano plots showed that the *COL24A1*, *TMEM252*, and *MARCO* genes were upregulated in fistula tissues, while *REG3G*, *CORO7‐PAM16*, and *ADGRF1* were downregulated (Figure 1c). *COL24A1* is involved in collagen remodeling, and its high expression is associated with extracellular matrix remodeling around intestinal fistulas.^[^ ^14^ ^]^ Overexpression of *TMEM252* inhibits cell migration and proliferation by affecting downstream signaling pathways.^[^ ^15^ ^]^ *MARCO* is associated with the regulation of the immune response to pathogens and inflammation, and its expression is upregulated due to localized infection and inflammation in patients with intestinal fistulas.^[^ ^16^ ^]^ Down‐regulated genes such as *REG3G*, *CORO7‐PAM16*, and *ADGRF1* are mainly involved in intrinsic immunity, regulation of actin dynamics, and suppression of inflammation in the intestine.^[^ ^17^ ^]^ This may result from the abnormal adhesion of the intestinal mucosa to the surrounding tissues at the intestinal fistula, as well as the disruption of the local mucosal barrier of the intestinal fistula, which is stimulated by intestinal fluids and intestinal microorganisms, causing a sustained inflammatory response.

Gene ontology (GO) and Kyoto Encyclopedia of Genes and Genomes (KEGG) analyses revealed that these DEGs were mainly involved in apoptosis, bacterial response, cell adhesion, actin regulation, and PI3K signaling pathways (Figure 1d,e). Gene set enrichment analysis further revealed the distribution characteristics of the DEGs (Figure S1, Supporting Information). The activity levels of the PI3K and MAPK signaling pathways, which are essential for cell growth, proliferation, differentiation, and survival, were inhibited in the intestinal fistula tissue.^[^ ^18^ ^]^ Reduced expression of REG3G may have attenuated the antimicrobial capacity of these tissues. Western blot analysis showed decreased levels of REG3G and phosphorylation of AKT and ERK in fistula tissues (Figure 1f,g), which corroborated our transcriptome results. These findings suggest that intensifying local antimicrobial treatments for intestinal fistulas and promoting cell proliferation to accelerate the re‐epithelialization of the intestinal mucosa may represent key strategies to promote their healing.

### 2.2 Synthesis and Characterization of Mechanically Active GNGP Hydrogels

**Figure 2:**
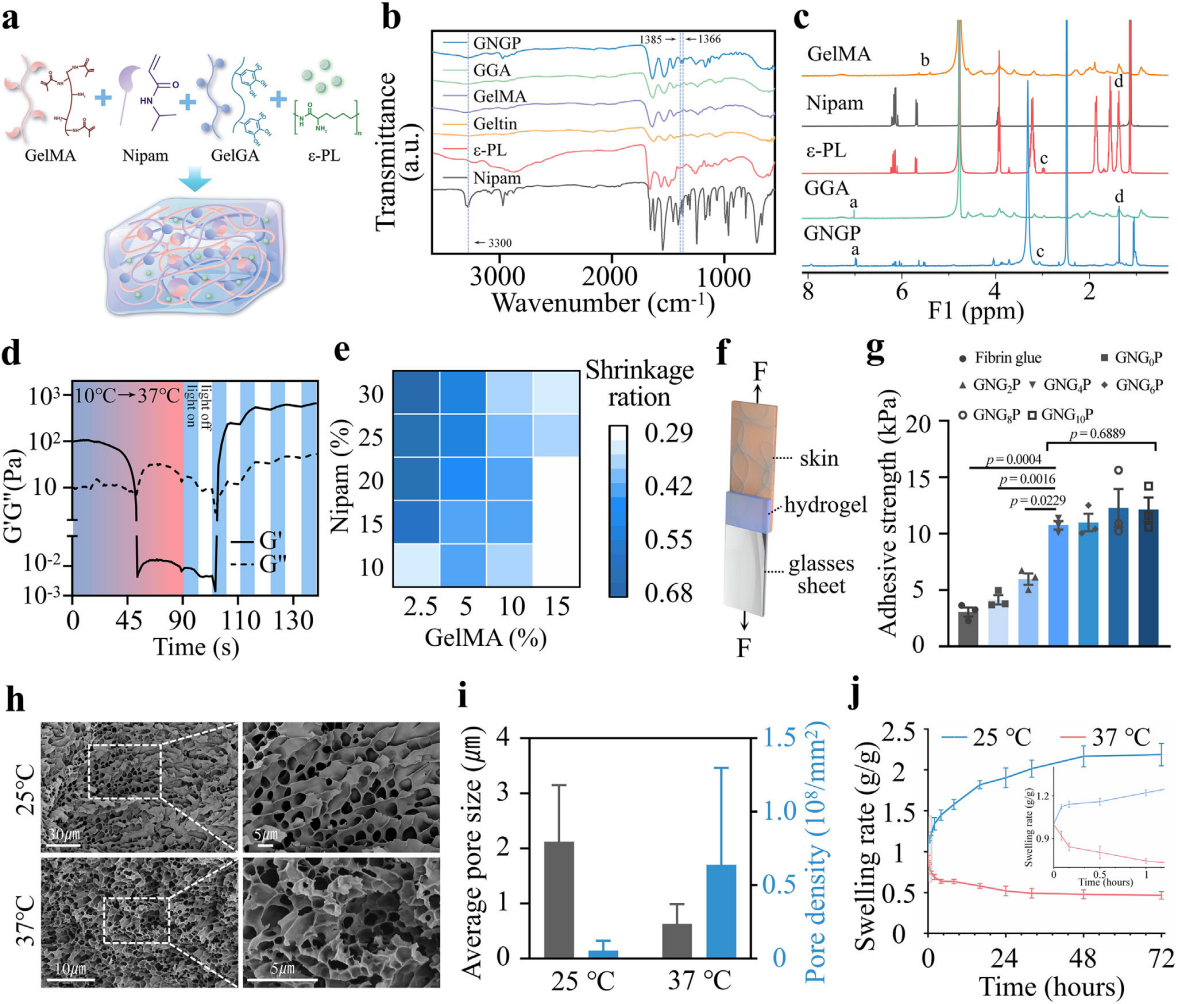
Synthesis and characterization of mechanically active GNGP hydrogels. a) Synthesis of GNGP hydrogels. b) Fourier‐transform infrared spectroscopy and c) NMR spectroscopy verified the synthesis of GNGP hydrogels. a: δ = 7.0 ppm; b: δ = 5.5 ppm, δ = 5.7 ppm; c: δ = 3.0 ppm; d: δ = 1.4 ppm. d) Variable temperature time sweeps and intermittent UV irradiation scans of GNGP hydrogel precursor solutions indicated the gel's initial state at a low temperature of 10 °C, which then rapidly transformed to a liquid state when the temperature was increased to 20 °C. e) Effects of different concentrations of GelMA and Nipam on the shrinkage properties of the hydrogel. f) Schematic diagram of experiments examining the adhesive properties of the GNGP hydrogel and fibrin glue under shear forces. g) GGA enhanced the adhesive performance of GNGP hydrogels, but the GGA proportion accounting for more than 4% did not offer significant further improvements (*n* = 3). h) Representative graphs of the pore size structures of the GNGP hydrogel at different temperatures through (i) quantitative analysis. The gel had a smaller pore size structure at 37 °C, which manifested macroscopically as a reduction in its volume (*n* = 3). j) The GNGP hydrogel underwent anomalous swelling at 37 °C as a result of hydrophobic forces squeezing out the internal fluid (*n* = 3). The *p*‐values in (g) are determined by one‐way ANOVA followed by Tukey's multiple comparisons test. Data are presented as mean ± SD.

Previous studies have shown that mechanically active hydrogels are effective for promoting wound healing by contracting and applying shear stress to wounds.^[^ ^19^ ^]^ However, these hydrogels are mostly prepared as patches, which do not adequately meet the requirements for sealing intestinal fistulas. Since intestinal fistula involves damage to the entire layer of skin, muscle and intestines, healing of skin wounds alone is often a pseudo‐healing process that is highly susceptible to recurrence. To overcome this drawback, mechanically active GNGP hydrogels with injectable properties were synthesized using the low‐temperature physical cross‐linking properties of gelatin (**Figure** **2**a). Fourier‐transform infrared (FTIR) spectral analysis revealed that the absorption peaks of Nipam and GelMA at 3300 cm^−1^ caused by the stretching vibration of the olefinic moiety were significantly attenuated in our GNGP hydrogel crosslinked by the photo‐crosslinking reaction, confirming the occurrence of the addition reaction. Quantitative nuclear magnetic resonance (NMR) spectroscopy further demonstrated that the GNGP hydrogel undergoes an addition reaction via photocrosslinking (Figure S2a,b and Table S1, Supporting Information). Meanwhile, the gel's absorption peaks at 1366 and 1385 cm^−1^ were attributed to the characteristic vibration of its isopropyl groups (Figure 2b).^[^ ^20^ ^]^ The gel's NMR spectrum showed proton signals at 7.0 ppm (a) for *o*‐phenyltriol, and at 5.5 and 5.7 ppm (b) for vinyl, confirming the synthesis of GGA with GelMA.^[^ ^12^, ^21^ ^]^ The grafting rates of 56.29% and 11.92% for GelMA and GGA, respectively, were assayed according to the method of Ovsianikov et al.^[^ ^22^ ^]^ (Figure S2c,d, Supporting Information).

The rheological properties of the GNGP hydrogel showed a rapid gel‐sol phase transition at body temperature and rapid curing under UV irradiation within 7 s to form a stable adhesion, which represents its ability to be injected and stay at the intestinal fistula, forming a topological adhesion interface with the local tissues under the effect of body temperature and rapidly sealing the intestinal fistula in situ under UV irradiation (Figure 2d). The modulus of intestinal and skin tissues was in the range of 1–10 kPa, and the GNGP hydrogel had a modulus similar to theirs, which facilitated the migration of intestinal mucosal epithelia and the repair of intestinal fistulas^[^ ^23^ ^]^ (Figure S3a, Supporting Information). The local temperature increased during UV irradiation and decreased after shutdown, resulting in a slight decrease in the storage modulus of the hydrogel. The stress–strain curves of the GNGP hydrogel were examined by a mechanical machine at 25 and 37 °C (Figure S3b,c,e,f, Supporting Information). The stress‐strain characteristics of GNGP hydrogel remained stable after being subjected to cyclic pressure of 50 kPa. This showed that the GNGP hydrogel had good elasticity and fatigue resistance, stabilizing itself against contraction and resisting environmental stresses during fistula sealing.

The shear stress generated by the mechanically active hydrogels depended on their deformability, cohesion, and interfacial adhesion strength. The deformability of GNGP hydrogels, which decreased with increasing GelMA concentration, was enhanced with increasing Nipam concentration (Figure 2e). This was mainly attributed to the hydrophobic collapse behavior of GNGP hydrogels with pNipam network structure resulting from conformational changes near 32 °C (Figure S3d, Supporting Information). The GelMA covalent network enhanced the stiffness but reduced the ductility of the hydrogels (Figure S4, Supporting Information). The GNGP hydrogels showed good deformability and cohesion when the concentration of Nipam was more than 20% and the concentration of GelMA was 5%. By testing the adhesive strength of the hydrogel under shear forces in pig skin and slides, we found that its adhesive strength was progressively enhanced as the GGA concentration increased, and when it reached 4%, the adhesion strength stabilized and was significantly stronger than that of the commercial adhesive (fibrin glue) (Figure 2f,g).^[^ ^24^ ^]^ Excellent adhesion properties ensure that the GNGP hydrogel seals the intestinal fistula for contraction and pulls the surrounding tissue to reduce the intestinal fistula defect area. The initial adhesion properties of the hydrogel without added GGA were mainly attributed to the hydrogen bonding at the hydrogel–tissue interface.^[^ ^25^ ^]^ Hydrogen bonding, cation–π interactions, and covalent bond formation at the interface after the addition of GGA enhanced the adhesion properties of the hydrogel.^[^ ^26^ ^]^ When the concentration of GGA was further increased, the adhesion strength of the GNGP hydrogels was not significantly enhanced, which may be related to the saturation of the interfacial cross‐linking (Figure S5, Supporting Information).

Scanning electron microscopy (SEM) confirmed the microstructural changes of the GNGP hydrogel during the contraction process (Figure 2h,i). The results of swelling experiments showed that the GNGP hydrogel exhibited a rapid and paradoxical swelling behavior at 37 °C, which was mainly caused by its internal hydrophobic forces that hindered the entry of water molecules (Figure 2j). The paradoxical swelling of GNGP hydrogel at body temperature facilitates the reduction of the size of the intestinal defect after sealing an intestinal fistula, avoids compression of the surrounding tissues after swelling, improves the migration path of intestinal epithelial cells, and accelerates the healing of the mucosa.^[^ ^27^ ^]^ It also extruded the internal liquid through its own contraction during application, forming a protective mucus layer on the surface that temporarily blocked the erosion of the intestinal fluid on the tissues and played a protective role.^[^ ^28^ ^]^

### 2.3 Sealing and Degradation Properties of GNGP Hydrogels

**Figure 3:**
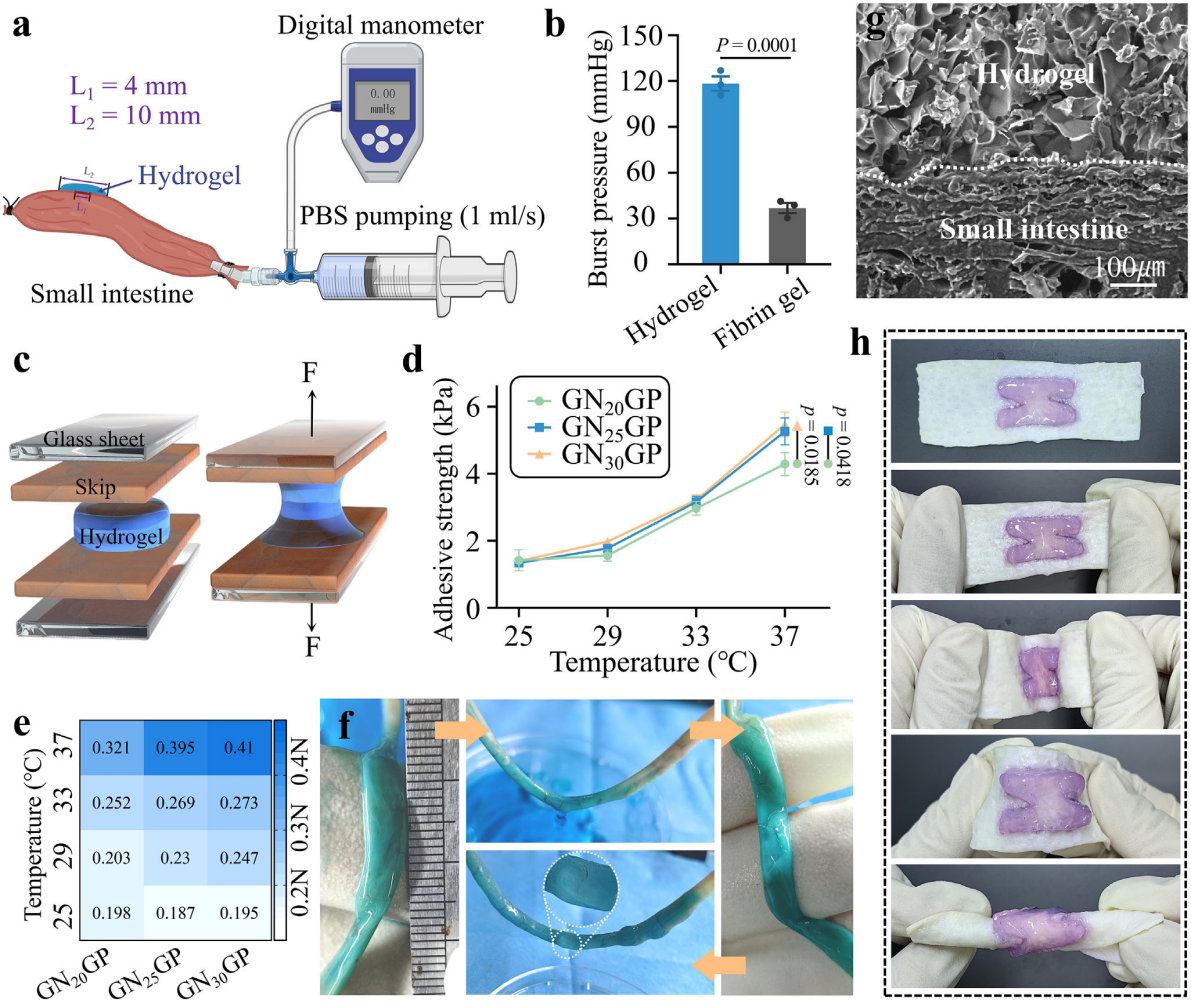
Sealing properties and mechanical activity of GNGP hydrogel. a) Schematic diagram of our experiment to determine the burst pressure of GNGP hydrogel when sealing an isolated human small intestine. b) The burst pressure of the GNGP hydrogel was significantly higher than that of fibrin glue (*n* = 3). c) Schematic diagram of a GNGP hydrogel adhesion test. d) The gel's adhesive strength correlated positively with Nipam concentration until >25%. e) Heat map of the adhesive strength of the GNGP hydrogel at different temperatures (*n* = 3). f) Schematic of the GNGP hydrogel sealing isolated intestinal tubes. For an 8 mm rat small intestinal injury parallel to the long axis, injected fluid no longer leaked from the injury after it was sealed using our mechanically active GNGP hydrogel. g) The GNGP hydrogel adheres tightly to an isolated human small intestine. h) The GNGP hydrogel forms a stable adhesion and resists multiple deformations. The *p*‐values in (b) and (d) are determined by two‐sided unpaired *t*‐test and one‐way ANOVA followed by Tukey's multiple comparisons test, respectively. Data are presented as mean ± SD.

The bursting pressure of our GNGP hydrogel was evaluated using isolated human small intestines (**Figure** **3**a). The bursting pressure was found to be 118.4 ± 8.2 mmHg, which was significantly higher than that of fibrin glue, a comparable standard clinical product (Figure 3b), and was able to adapt to the intestinal pressure. Higher mechanical strength ensures that the hydrogel remains stable under intestinal movement and pressure, preventing hydrogel rupture due to mechanical damage. The mechanical activity of the GNGP hydrogel was indirectly assessed through variable temperature adhesion testing, which determined the optimal concentration of Nipam to be 25% for subsequent experiments, because further increases in Nipam concentration did not significantly increase the gel's adhesive strength (Figure 3c–e). Video S1 (Supporting Information) shows that the mechanically active GNGP hydrogel was able to close abdominal wall defects in rats at 37 °C. It was also able to effectively seal isolated small intestinal leakages in rats (Figure 3f). Its microstructure confirmed the formation of a tight interfacial bond between the hydrogel and human small intestinal tissues, indicating that the gel was capable of stably adhering to intestinal tissues (Figure 3g).

The GNGP hydrogel exhibited excellent deformation resistance when subjected to external forces (Figure 3h). After immersion in simulated body fluids and simulated intestinal fluids for 24 h, the GNGP hydrogel maintained its deformation resistance in a liquid environment (Figure S6, Supporting Information). In addition, GNGP hydrogel was able to adhere stably to the simulated peristaltic interface of porcine small intestine in PBS (Video S2, Supporting Information). This demonstrates that GNGP hydrogels are able to adapt to intestinal peristalsis and complex movements of body parts. The gel showed good adhesion to tissues such as the heart, liver, spleen, and other important organs (Figure S7, Supporting Information), which implies the possibility of favorable sealing functions in other clinical scenarios as well. The GNGP hydrogel was able to resist digestion with intestinal fluids in the short term, but it was difficult to resist long‐term sustained intestinal fluid stimulation (Figure S8, Supporting Information). This was mainly due to the fact that the simulated intestinal fluid was rich in digestive enzymes that hydrolyzed the gelatin in the GNGP hydrogel. Since only one side of the GNGP hydrogel was in contact with the intestinal fluid as it flowed through the fistula and the adhesion interface was relatively stable in the intestinal fluid. Therefore, GNGP hydrogel is relatively stable during fistula sealing. After hydrogel contraction, the few hydrogels protruding outside the fistula are cut off to avoid impeding IEC migration under continuous intestinal fluid stimulation.

**Figure 4:**
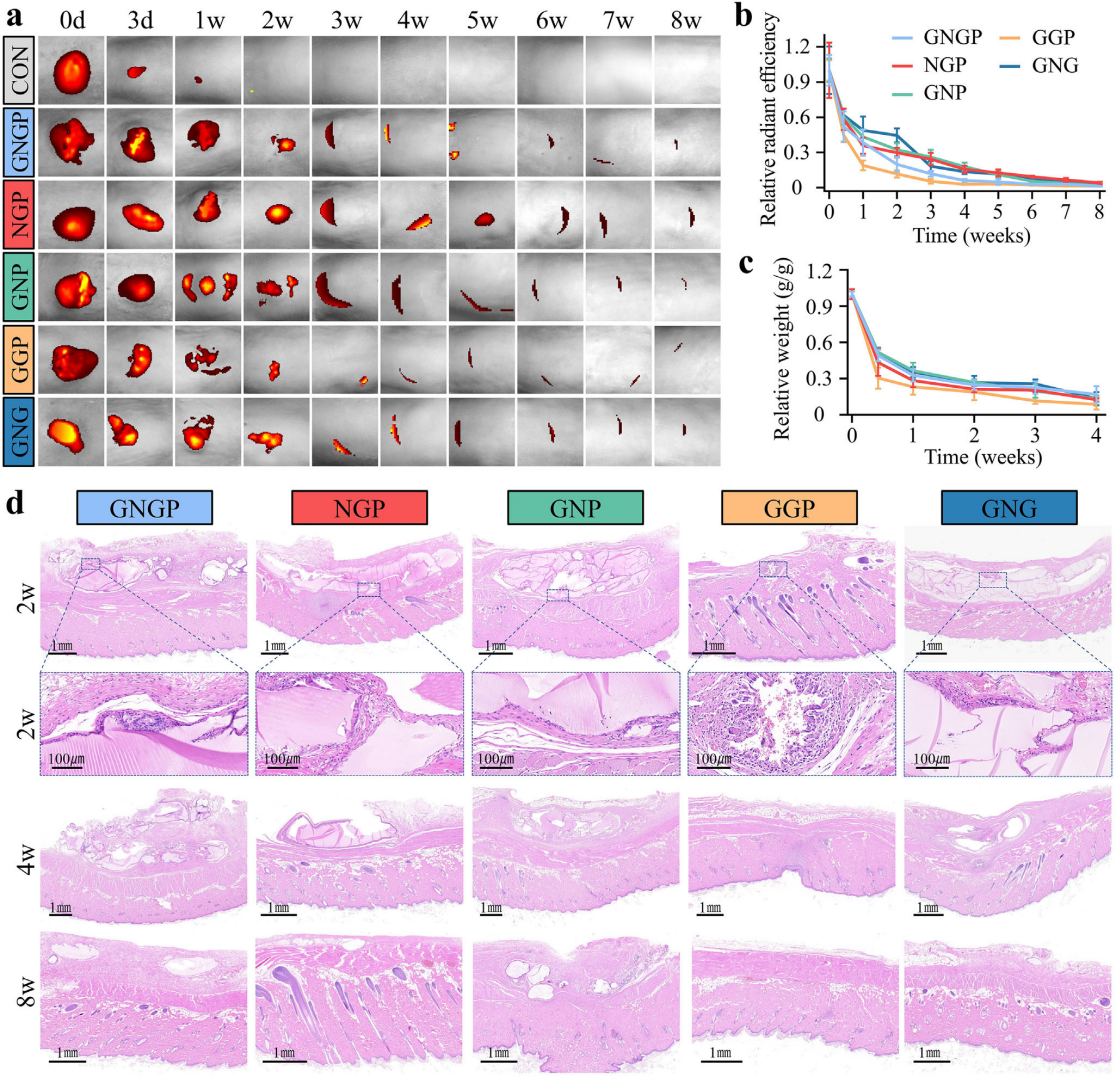
Mechanically active GNGP hydrogel with suitable degradation properties and histocompatibility. a) Living imaging of GNGP hydrogels embedded in rat subcutis tissues with grafted fluorescent probes, and b) quantitative analysis (*n* = 3). The degradation time of the GNGP hydrogel was extended by the addition of Nipam. c) Quantitative analysis of the weight of GNGP hydrogels embedded in rat subcutis after removal and lyophilization (*n* = 3). d) The GNGP hydrogel showed excellent histocompatibility. After the subcutaneous implantation of the hydrogel into the backs of SD rats, cells crawled along its surface without triggering significant foreign body inflammatory reactions.

Degradability and histocompatibility are essential factors for using sealing materials in intestinal fistula treatment. A fluorescent Cy5.5 probe conjugated to the hydrogel was used to track its degradation.^[^ ^29^ ^]^ After eight weeks, all the hydrogel groups exhibited up to 95% degradation in rats (**Figure** **4**a,b). Among them, the hydrogel without the Nipam fraction degraded the fastest, with a degradation rate about 80% in the first week, compared with 60% for the GNGP hydrogel. The degradation rate of GNGP hydrogel is favorable for mucosal healing after sealing the intestinal fistula. The GNGP hydrogel provides a scaffold for the migration of IECs at the initial stage, while being able to degrade to avoid local tissue discomfort and chronic inflammation. The degradation of the hydrogel over four weeks of subcutaneous embedding in rats as measured by weighing followed the same trend as the fluorescent tracking assay (Figure 4c). We further elucidated the biological responses associated with hydrogel degradation and subsequent tissue substitution by hematoxylin and eosin (H&E) staining of local tissues. The results showed that the GNGP hydrogel had good histocompatibility in vivo, caused few foreign body inflammatory reactions, and provided an ideal interface for cell migration (Figure 4d). In addition, its degradation did not cause any significant damages to vital organs, indicating that it had satisfactory safety when used in vivo (Figure S9, Supporting Information). Previous studies have shown that the potential cytotoxicity of Nipam after polymerization reaction mainly comes from the unreacted or degraded monomers that may be present during the preparation process, and the concentration of Nipam monomers higher than 0.5 mg mL^−1^ for 3 h leads to cytotoxicity, which is mainly manifested as apoptosis.^[^ ^30^ ^]^ Degradation experiments of the GNGP hydrogel in simulated body fluids or simulated intestinal fluids have shown that the cumulative concentration of the Nipam monomers that were decomposed in the 24 h was less than 0.2 mg mL^−1^ (Figure S10, Supporting Information). This fraction of monomers was rapidly diluted in the intestinal lumen, resulting in the concentration of Nipam much lower than the cytotoxic dose. In addition, the apoptosis of surrounding cells after subcutaneous encapsulation of GNGP was evaluated by TUNEL staining and Caspase3 staining (Figure S11, Supporting Information). No significant apoptosis was found to occur in cells directly exposed to the GNGP hydrogel. Therefore, GNGP has a favorable in vivo biosafety.

### 2.4 Cytocompatibility and Antimicrobial Properties of GNGP Hydrogels

**Figure 5:**
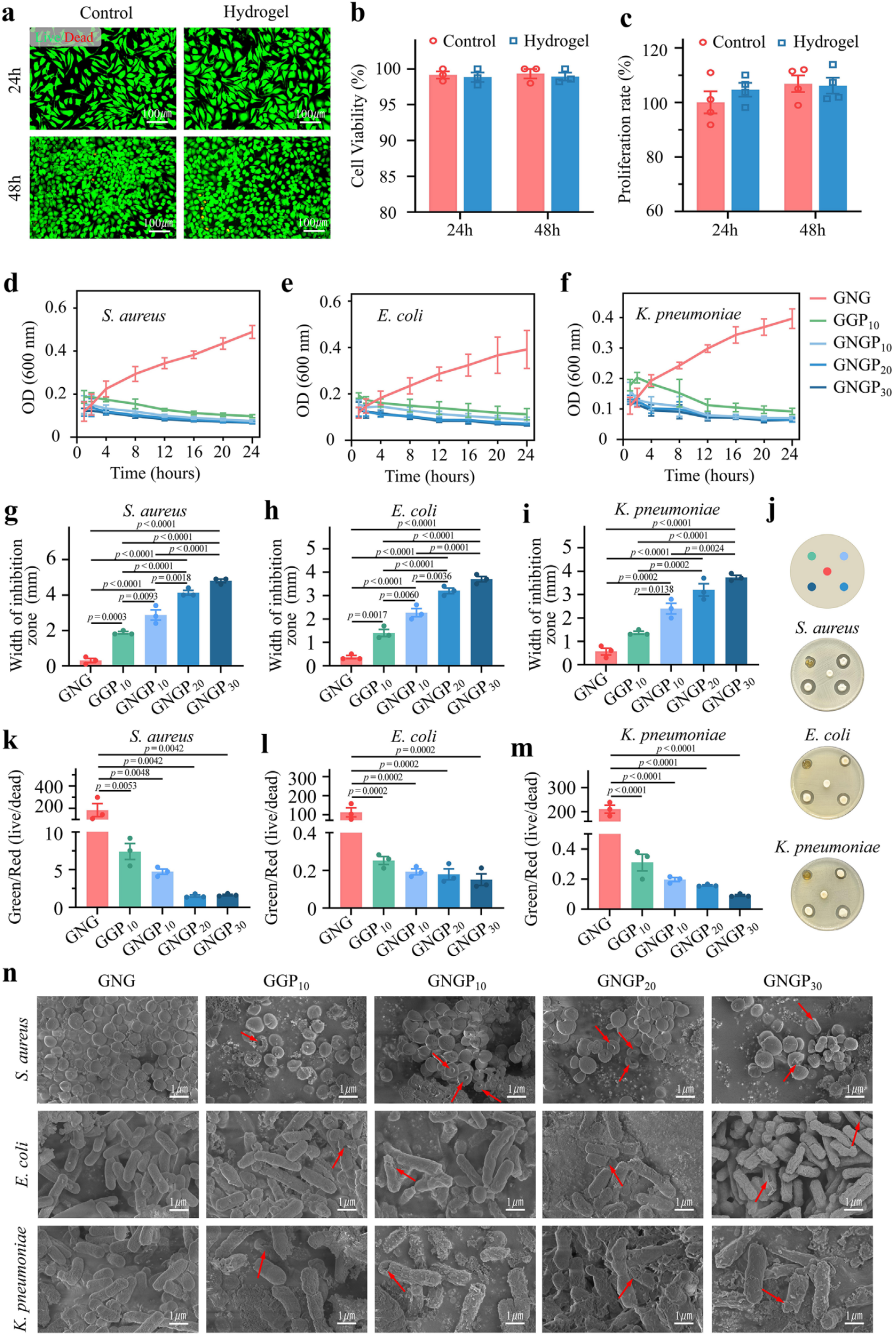
Mechanically active GNGP hydrogels with excellent cytocompatibility and antimicrobial properties. a) Live/dead cell staining and b) quantitative analysis of L929 fibroblasts co‐cultured with our GNGP hydrogel (*n* = 3). c) Quantitative analysis of a CCK‐8 assay of L929 fibroblasts co‐cultured with our GNGP hydrogel (*n* = 3). d–f) OD600 of *Staphylococcus aureus*, *Escherichia coli*, and hyper‐virulent *Klebsiella pneumoniae* co‐cultured with different hydrogels: quantitative analysis (*n* = 3). g–j) Bacteriological zones produced by different hydrogels. Quantitative analysis of inhibition zones (*n* = 3). k–m) Quantitative analysis of live/dead staining of pathogenic bacteria after 8 h of co‐culture with hydrogels (*n* = 3). n) Morphological changes in pathogenic bacteria after co‐culturing with hydrogels. Red arrows mark the morphological abnormalities in the bacteria. The *p*‐values in (b, c) and (g)–(m) are determined by two‐sided unpaired *t*‐test and one‐way ANOVA followed by Tukey's multiple comparisons test, respectively. Data are presented as mean ± SD.

To fully assess the biosafety of our GNGP hydrogel, rat fibroblasts were incubated in media containing its extract for 24 and 48 h. The percentage of viable cells was >97%, which did not differ significantly from that of the control group (**Figure** **5**a,b). This indicated that the GNGP hydrogel did not have an acute toxic effect on the cellular activity. This was also confirmed through a CCK‐8 assay (Figure 5c). Meanwhile, intestinal epithelial cells and fibroblasts did not show significant apoptosis after 24 h of incubation in the medium containing GNGP hydrogel extract (Figure S12, Supporting Information). After co‐incubation of the hydrogel and its precursor solution with rat erythrocytes, the concentration of hemoglobin in the supernatant showed no significant erythrocyte cytolysis, which suggests good hemocompatibility (Figure S13a, Supporting Information). Therefore, we concluded that our mechanically active GNGP hydrogel is suitable for the clinical treatment of intestinal fistula.

The antibacterial properties of the hydrogel were evaluated using common clinically pathogenic bacterial strains with different cell wall structures and levels of virulence—including *Staphylococcus aureus*, *Escherichia coli*, and the highly virulent *Klebsiella pneumoniae*. The growth rates of the bacteria when co‐cultured with the hydrogel were detected using spectrophotometry at an absorbance frequency of 600 nm, and their survival statuses were assessed by measuring the size of the inhibition zone, the count of viable colonies, and live versus dead staining (Figure 5d–m and Figure S13b,c, Supporting Information). Finally, the morphological structures of the bacteria were observed via SEM (Figure 5n). The results showed that the gel possessed superior antibacterial properties, which were enhanced with increasing concentrations of ε‐PL. Notably, the growth rate of the highly virulent *K. pneumoniae* was transiently elevated in the low ε‐PL group, before later being inhibited after 2 h of incubation. This was thought to be related to its thicker pod membrane structure. In the hydrogel group, the growth of *K. pneumoniae* was significantly inhibited after 1 h of incubation, suggesting that the gel's mechanical activity promoted the effective release of ε‐PL and thus enhanced its antibacterial effect. Considering its antimicrobial efficacy, we chose an ε‐PL concentration of 20% for our subsequent experiments.

### 2.5 Mechanically Active GNGP Hydrogel Promotes Healing of Intestinal Fistula

**Figure 6:**
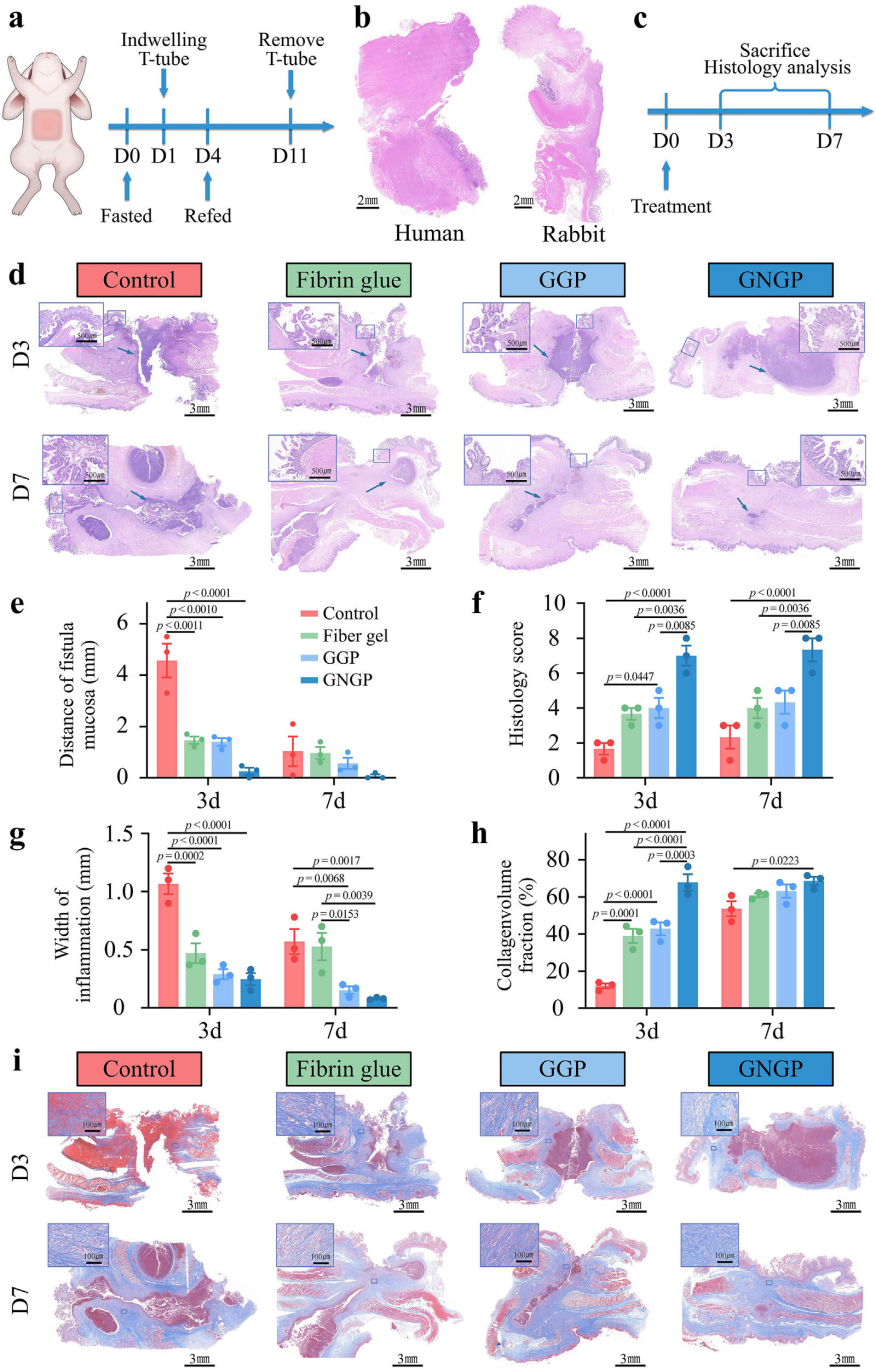
GNGP mechanically active hydrogel induced repair of intestinal fistulas in a rabbit model. a) Experimental procedures for constructing a rabbit intestinal fistula model. b) H&E staining of clinical intestinal fistula samples and local tissues of our rabbit model of intestinal fistula. Adhesion of the intestinal fistula to the abdominal wall. c) Experimental procedures for the treatment of rabbit intestinal fistulas. d) Representative H&E staining of tissues around the fistula after 3 and 7 d of treatment using different methods. e) Quantitative analysis of the distance of intestinal mucosa at the internal fistula, showing that the mechanically active GNGP hydrogel effectively reduced the intestinal mucosa distance on both sides of the fistula (*n* = 3). f) Pathological scoring of the local intestinal fistula tissue (n = 3). g) Quantitative analysis of the inflammation distance in the fistula tract (*n* = 3). h) Collagen volume fraction of the tissue surrounding the fistula tract (*n* = 3). i) Representative Masson staining of tissues around the fistula after 3 and 7 d of treatment using different methods. The *p*‐values in (e)–(h) are determined by two‐way ANOVA followed by Tukey's multiple comparisons test. Data are presented as mean ± SD.

To evaluate the potential of hydrogel for clinical applications related to intestinal fistula treatment, we simulated the pathophysiological process of intestinal fistula formation and successfully constructed a rabbit model of intestinal fistula using a scaffold‐embedding method (**Figures** **6**a and S14, Supporting Information). After the model was established, HE and immunofluorescence staining of the intestinal fistula revealed that the intestines formed adherent tissues with the abdominal wall (Figure 6b). A similar trend in phosphorylation levels of ERK and AKT was also observed in the intestines of the animal model and the intestinal fistula patients (Figure S15, Supporting Information). The flow of the animal experiments is shown in Figure 6c. HE staining after the third day of intestinal fistula treatment showed a reduction in the area of intestinal mucosal defects in all of the experimental groups (Figure 6d–f). However, the fistula in the blank control group showed a significant inflammatory reaction (Figure 6g). By contrast, the intestinal mucosa in the mechanically active GNGP hydrogel group was nearly fully healed. On the seventh day of treatment, the intestinal fistula in the mechanically active GNGP hydrogel group had achieved full‐layer healing. Masson staining revealed that more collagen fiber deposition was observed in the mechanically active GNGP hydrogel group after 3 d of treatment, which facilitated subsequent tissue reconstruction (Figure 6h,i). Ki67 and proliferating cell nuclear antigen (PCNA) are important markers of the proliferative state of cells to assess the regeneration of the intestinal mucosa.^[^ ^31^ ^]^ The expression of Ki67 and PCNA was significantly increased in the GNGP group, indicating that the proliferation of intestinal mucosal epithelial cells was accelerated after GNGP hydrogel treatment (Figure S16, Supporting Information). The healing of the intestinal mucosa is crucial for repairing intestinal injuries, and the efficacy of our mechanically active GNGP hydrogel was mainly attributed to its contractile action. This property effectively reduced the size of the fistula, resisted the bacteria and intestinal fluid output, and provided support for the proliferation and migration of IECs.

### 2.6 Molecular Features Related to Intestinal Fistula Healing

**Figure 7:**
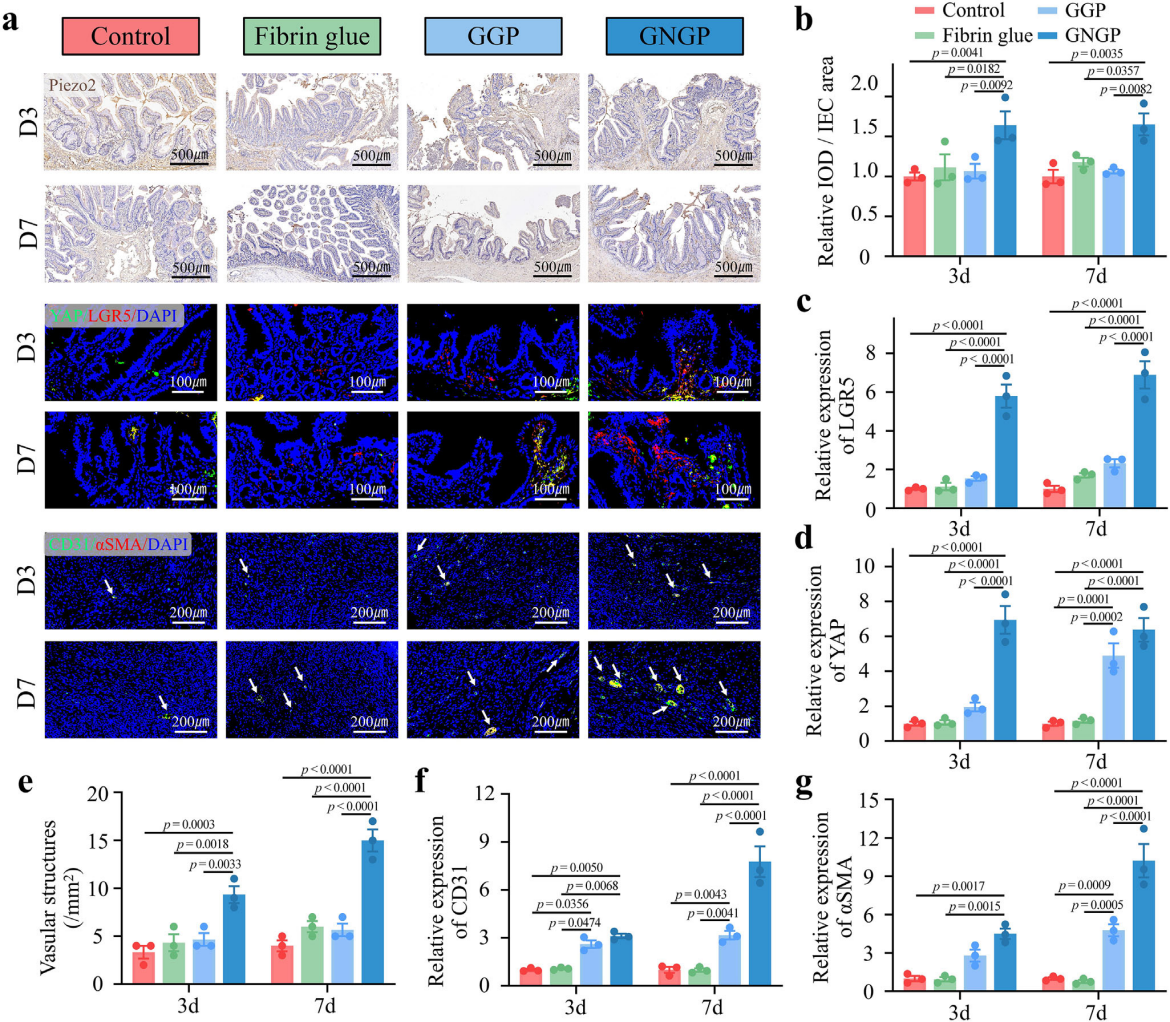
GNGP mechanoactive hydrogel modulates the mechanosensitivity of intestinal mucosal epithelial cells in intestinal fistula. a) Representative images of immunohistochemistry of Piezo2 and immunofluorescence of YAP, LGR5, CD31, and αSMA in intestinal fistula. White arrows indicate neovascularization. b) Quantitative analysis of Piezo2 in intestinal fistulas treated with GNGP hydrogel (*n* = 3). c) Quantitative analysis of LGR5 expression in intestinal fistula and d) YAP expression. GNGP hydrogel modulates the mechanosensitivity of intestinal mucosal epithelial cells (*n* = 3). e) Density of neovascularization in perifistula tissues (*n* = 3). f) Quantitative analysis of CD31 expression and g) αSMA expression in perifistula tissues. GNGP hydrogel promotes perifistula blood vessel formation (*n* = 3). The *p*‐values in (b)–(g) are determined by two‐way ANOVA followed by Tukey's multiple comparisons test. Data are presented as mean ± SD.

To further determine the effect of the mechanically active hydrogel on intestinal fistula healing, we performed immunostaining of intestinal fistula tissues during healing (**Figure** **7**a). Piezo2, a mechanosensitive ion channel, is a proprioceptive and tactile receptor that regulates intestinal motility.^[^ ^32^ ^]^ In the GNGP hydrogel group, the expression level of the Piezo2 was found to be significantly increased, suggesting that it may be involved in the intestinal recognition of shear stress (Figure 7b). YAP is a key molecule for mechanical signal transduction that affects intestinal epithelial developmental initiation and regeneration.^[^ ^33^ ^]^ LGR5 is a marker for intestinal stem cells.^[^ ^34^ ^]^ We found a significant increase in the expression levels of both YAP and LGR5, particularly in healing intestinal fistula tissues that were treated with GNGP hydrogel (Figure 7c,d). The formation of microvessels represents another important indicator for assessing the effect of tissue repair. By immune‐labeling the tissues with CD31 and αSMA, we observed that the density of microvessels in the GNGP hydrogel group was significantly higher than that in the control one, which further corroborated the promoting effect of the gel in terms of intestinal fistula repair (Figure 7e–g).^[^ ^35^ ^]^

To reveal the changes in gene expression following tissue treatment with our mechanically active hydrogel, we performed RNA sequencing on healing intestinal fistula tissues after GNGP hydrogel treatment (Figure S17a,b, Supporting Information). The DEGs in the two groups were mainly enriched in biological processes such as the inflammatory response, cell signaling, and the MAPK signaling pathway (Figure S17c, Supporting Information). Immunofluorescence assay results further verified that the phosphorylation level of ERK, a key protein in the MAPK signaling pathway, showed a significant increase in the intestinal fistula healing tissues under the action of the hydrogel, which was consistent with our RNA sequencing results (Figure S17d,e, Supporting Information). These results indicated that the mechanically active hydrogel upregulated the expression level of YAP in the IECs and enhanced their stem cell properties. Moreover, the MAPK pathway was involved in the promotion of intestinal fistula healing by the mechanically active hydrogel.

### 2.7 Shear Stress Facilitates IEC Proliferation through YAP

**Figure 8:**
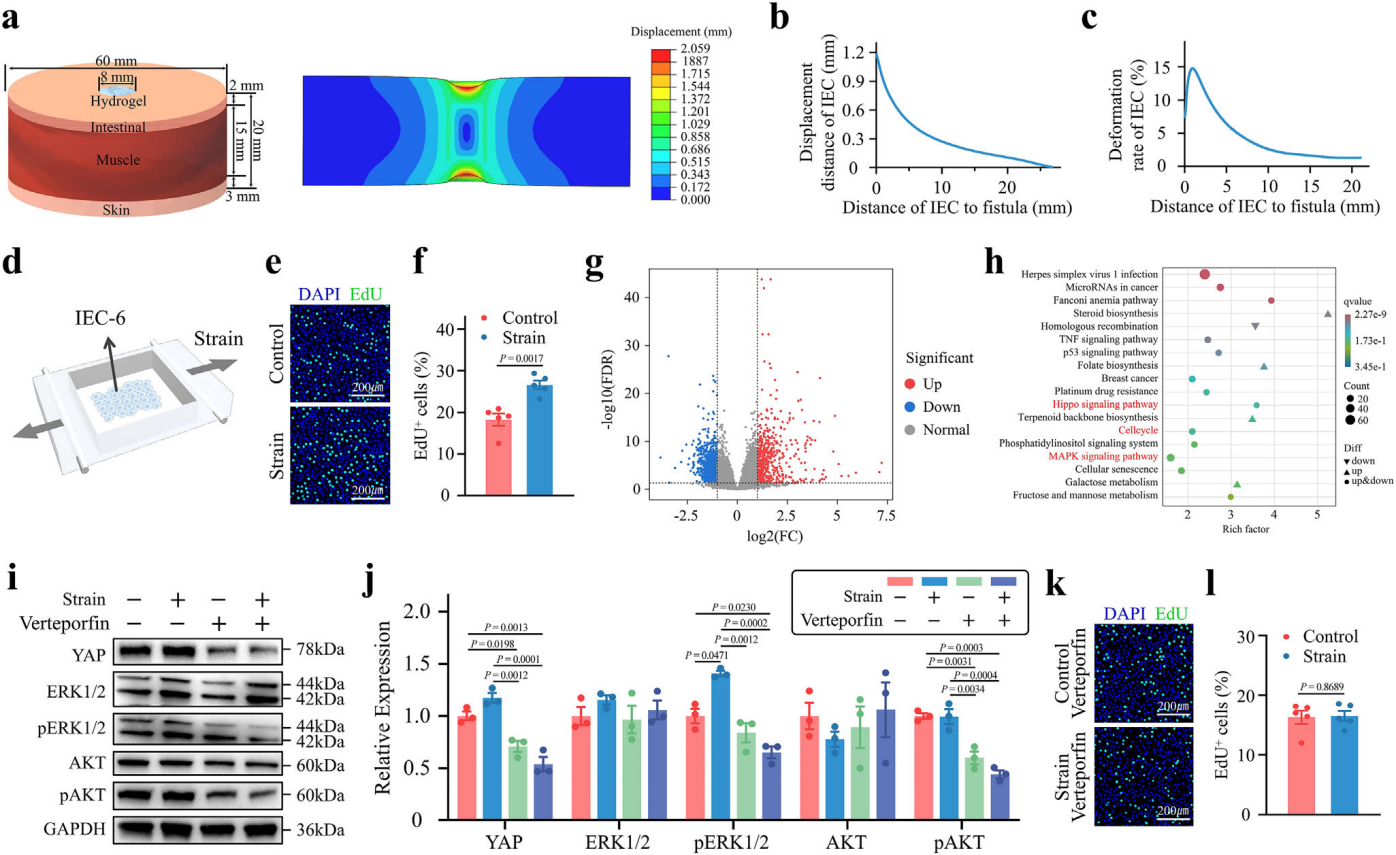
YAP activation following strain stretching promotes IEC proliferation. a) Schematic and results of a finite element analysis of the GNGP hydrogel sealing intestinal fistulas during treatment. b,c) Simulation of the displacement and deformation of intestinal fistulas after GNGP hydrogel sealing treatment. d) Schematic diagram of the experimental platform for conducting mechanical stimulation using stretching chambers. e) EdU staining and f) quantitative analysis of IECs after shear stress stimulation for 4 h. The number of proliferating IECs increased significantly after 4 h of shear stress stimulation (*n* = 5). g) Volcano plot of differentially expressed genes in shear stress‐stimulated versus unstimulated IECs, and h) KEGG analysis. i) WB bands and j) quantitative analysis after shear stress stimulation and application of vertiporfin, a pharmacological inhibitor of YAP. Vertiporfin effectively inhibited YAP expression in IECs. Shear stress promoted the phosphorylation of AKT and ERK in IECs, whereas this activation effect was suppressed when YAP was inhibited by vertiporfin (*n* = 3). k) EdU staining and l) quantitative analysis of IEC stimulated by shear stress for 4 h after YAP inhibition by verteporfin. There was no significant effect of shear stress on the proliferation of IECs once YAP was inhibited (*n* = 5). The *p*‐values in (f, l) and (j) are determined by two‐sided unpaired *t*‐test and one‐way ANOVA followed by Tukey's multiple comparisons test, respectively. Data are presented as mean ± SD.

The mechanical stimulation exerted by the GNGP hydrogel on the intestinal mucosa was analyzed through finite element simulation of how it sealed an intestinal fistula (**Figure** **8**A–C). Through this, we constructed a shear stress stimulation platform for the cells (Figure 8D). On this platform, we applied a shear stress stimulus to the IECs to simulate the actual mechanical effects produced by the GNGP hydrogel during intestinal fistula treatment. The proliferating cells were tracked via EdU staining, which revealed that the number of proliferating cells increased significantly after the application of a stretch force (Figure 8E,F), indicating that stretching had a positive effect on IEC proliferation. Further transcriptomic analysis revealed significant changes in gene expression within IECs after shear stress was applied (Figure 8G). Through a KEGG analysis (Figure 8H), we found that these DEGs were mainly concentrated in the cell cycle, Hippo, and MAPK signaling pathways. Figure 8I,J shows that the expression or phosphorylation levels of core effector molecules in these pathways were reduced.

To further explore the role of YAP in shear stress‐stimulated IEC proliferation, we treated the cells with vitexoporfin, a pharmacological inhibitor of YAP. With YAP inhibition, the promotion of IEC proliferation by shear stress was reduced, and the phosphorylation levels of both AKT and ERK were significantly decreased (Figure 8I–L). We thus concluded that our mechanically active GNGP hydrogel released shear stress to mediate the proliferation of IECs through YAP, thus promoting intestinal fistula healing.

## 3 Conclusion

Intestinal fistula is a serious, life‐threatening condition that currently lacks effective closure measures to promote healing. Based on a biologically driven design concept, an antimicrobial GNGP hydrogel with mechanically active and injectable properties was developed to accelerate the healing of intestinal fistulas. This hydrogel was able to effectively seal intestinal fistulas and promote the healing process using contraction that was triggered by body temperature. The hydrogel also bridged IEC defects across intestinal fistulas and enhanced intestinal stemness by applying shear stress on IECs, which ultimately promoted the functional repair of intestinal fistulas. Mechanistically, we found that shear stress promoted IEC proliferation through YAP‐mediated mechanosensitization, and reversed low expression levels of proteins in the PI3K and MAPK signaling pathways within intestinal fistula tissues. While additional clinical applications may be possible, they were not investigated in this study. In summary, the design of a GNGP hydrogel for the promotion of intestinal fistula healing may enable a range of applications for potential fistula conditions such as esophageal fistula, pancreatic fistula, and rectovaginal fistula.

This study had several limitations. First, to avoid severe infections leading to death of experimental animals, we limited the degree of infection. Although the effectiveness of the mechanically active hydrogel was evaluated in this study through a rabbit intestinal fistula model, future experiments will focus on evaluating the effectiveness in omnivorous mammals such as pigs and intestinal fistula patients. In addition, although the biocompatibility of the mechanically active hydrogel in vivo and ex vivo was ideal. However, the healing process of intestinal fistula may still be subject to foreign body reaction. We should consider optimizing the hydrogel in the future to enhance its antidigestive properties and accelerate its degradation in vivo for better application in intestinal fistula sealing treatment.

## 4 Experimental Section

### Materials

Methacrylic anhydride (MA), gelatin, Nipam, ε‐PL, and 2‐hydroxy‐4′‐(2‐hydroxyethoxy)‐2‐methylpropiophenone (I2959) were purchased from Sigma‐Aldrich (St. Louis, MO). Gallic acid (GGA) and simulated intestinal fluid were purchased from Macklin Inc. (Shanghai, China). Cy5.5‐NHS ester was purchased from Duofluor Inc. (Hubei, China). 5‐Ethynyl‐2‐deoxyuridine (EdU) was purchased from Wuhan Servicebio Technology Co., Ltd. (Hubei, China). Live/Dead Cell Staining Kit, CCK‐8 Kit, and SYTO dyes were purchased from KeyGEN BioTech (Jiangsu, China). One step TUNEL Apoptosis Assay Kit was purchased from Beyotime Biotechnology (Shanghai, China). Verteporfin was purchased from Aladdin Co., Ltd. (Shanghai, China). Anti‐ERK (ab184699), anti‐Phospho‐ERK (ab201015), and anti‐Phospho‐AKT (ab131443) were purchased from Abcam (Cambridge, MA). Anti‐YAP1 (SAB1404823) was purchased from Sigma‐Aldrich (St. Louis, MO). Anti‐AKT (A24477), anti‐Phospho‐AKT (A24477), anti‐LGR5 (A10545), anti‐REG3G (A2146), and anti‐GAPDH (AC002) were purchased from Abclone (Wuhan, China). Anti‐Piezo2 (26205‐1‐AP) was purchased from Proteintech (Chicago, IL). Anti‐CD31 (GB120005), anti‐αSMA (GB13044), and Anti‐Caspase3 (GB11009) were purchased from Wuhan Servicebio Technology Co., Ltd. (Hubei, China). Commercial fibrin glue (Porcine Fibrin Sealant Kit) was produced by Beixiu Biotechnology Co., Ltd., Guangzhou, China. All other reagents were of analytical reagent grade.

*GelMA*: GelMA was synthesized according to a previously described protocol.^[^ ^12^ ^]^ Briefly, gelatin (8 g) was dissolved in phosphate‐buffered saline (PBS; 100 mL, 8 w/v%) and stirred at 60 °C for 3 h. MA (3 mL, 20.1 mmol) was then added dropwise at a rate of 0.3 mL min^−1^, after which the reaction proceeded at 55 °C for 3 h. The reaction was terminated by the addition of PBS (100 mL) and dialyzed using a dialysis membrane with a molecular weight cut‐off of 12–14 kDa for 7 d, during which time the deionized water was changed three times per day. The dialyzed solution was then eventually lyophilized.

*GGA*: Based on previously reported protocols,^[^ ^36^ ^]^ gelatin (5 g) was dissolved in deionized water (150 mL) at 40 °C. GA (3.4 g) was dissolved in a mixture of N,N‐dimethylformamide (DMF; 75 mL) and deionized water (50 mL). Subsequently, N‐hydroxy succinimide (3.2 g, 27.8 mmol) and 1‐(3‐dimethylaminopropyl)‐3‐ethylcarbodiimide (3.8 g, 24.5 mmol) were added to the GA solution and stirred for 1 h at room temperature. The GA solution was added to the gelatin solution and stirred overnight at 40 °C. Dialysis was performed for 3 d using a dialysis membrane with a molecular weight cut‐off of 12–14 kDa, and the deionized water was changed three times per day. The dialyzed solution was then lyophilized.

### Preparation of GNGP Hydrogels

GelMA, GGA, Nipam, and ε‐PL were dissolved in PBS containing I2959 (0.5 w/v%) to obtain solutions of different concentrations. These solutions were then mixed in a 3:3:3:1 volume ratio to form a homogeneous precursor solution. The mixed solutions were placed in a 4 °C environment and exposed to UV light (≈6.0 W cm^−2^) to initiate a polymerization reaction to form the GNGP hydrogel. The same method was used to prepare GNG, GNP, GGP, and NGP hydrogels—with the difference that only three solutions were involved in the mixing. The mixing ratios were the same as those used for the GNGP hydrogel. The concentrations of the components are detailed in Table S2 (Supporting Information).

### Materials Characterization

Gelatin, Nipam, ε‐PL, and lyophilized samples of GelMA, GGA, and GNGP were ground into fine powders. The samples were analyzed via spectral scanning using the KBr pellet method on a Nicolet‐6700 spectrometer (Thermo‐Fisher Scientific, Waltham, MA). The synthesis of GelMA, GGA, and GNGP was confirmed via NMR spectroscopy. Nipam and ε‐PL, and lyophilized samples of GelMA, GGA, and before and after UV irradiation of GNGP were placed in 5 mm NMR sample tubes with 0.6 mL of fully dissolved deuterium oxide to measure the ^1^H NMR spectrum (Bruker; Karlsruhe, Germany). Quantitative NMR was performed using maleic acid as an internal standard. The rheological properties of the GNGP hydrogels were measured using a rheometer (MCR302; Anton Paar GmbH, Graz, Austria). The distance between parallel plates was set to 1 mm. In the variable‐temperature oscillation time sweep experiment, the temperature was increased from 10 to 37 °C at a rate of 20 °C min^−1^, the strain was set to 1%, and the frequency was fixed at 10 Hz. In the alternating UV‐illuminated rheological time sweep, the GNGP precursor solution was subjected to five cycles of UV irradiation and switching off at 25 °C, with each irradiation and switching off period lasting 5 s. In the frequency sweep experiments of oscillation, the constant strain was set to 1%. The morphological structures of the GNGP hydrogel at 25 and 37 °C were observed via SEM (Phenom, Eindhoven, Netherlands).

To evaluate the swelling characteristics of the hydrogels, the mass (*M*_I_) of a hydrogel with a volume of 1 cm^3^ was weighed and immersed in 5 mL of PBS at 25 or 37 °C. The hydrogels were removed at specific time points and their surfaces were wiped to remove moisture. The weight (*M*_T_) of each sample was weighed and recorded before it was returned to the original PBS. The swelling rate (SR) was calculated using the formula:

(1) $$\mathrm{SR}=\frac{M_{T}-M_{I}}{M_{I}}\times 100\%$$

Differential scanning calorimetry (DSC) was performed using a simultaneous thermal analyzer (STA 449 F3; Netzsch, Bavaria, Germany). The weight of each sample was 10 mg. The temperature detection range was set to 15–50 °C, and the scanning rate was 2 °C min^−1^ for scanning.

### Mechanical Activity and Adhesive Properties

Hydrogels with different ratios were prepared as cylinders with a diameter of 10 mm and a height of 3 mm. These were placed in a water bath at 37 °C, and the change in diameter was measured to assess the hydrogel's deformability. The deformation rate was calculated using the formula:

(2) $$\mathrm{DR}=\left(1-\frac{L^{2}}{L_{0}^{2}}\right)\times 100\%$$

where DR is the deformation rate, *L*_0_ is the initial diameter, and *L* is the final diameter.

The hydrogel samples were prepared as cylindrical shapes with a diameter of 10 mm and a length of 6 mm. The preload of the universal mechanical machine (MTSCMT2103; MTS Systems, Eden Prairie, MN) was set at 0.05 N. The compression rate was 10 mm min^−1^ and the stress value in the cyclic test was 80% of the maximum strain.

The adhesive properties of the hydrogels were examined using a universal mechanical machine. Fresh pig skin was cut into 3.5 × 1 cm pieces of 2 mm thickness, to which hydrogel precursor solution (200 µL) was added dropwise. Microscope slides were then covered with a contact area of 1 cm^2^ between the slide and the pig skin, and were irradiated with UV light for 20 s at 4 °C to solidify the hydrogel. Adhesion measurements were performed by carefully fixing the slide and pigskin on a universal testing machine at 25 °C.

For the thermos‐stimulation adhesion (tensile fracture) assay, cylindrical hydrogel samples with diameters of 10 mm and heights of 2 mm were placed between two pieces of fresh pig skin and irradiated with UV light to form the hydrogels. The slides with the fixed pig skins were then acclimatized to the ambient temperature for 30 min at 25, 29, 33, or 37 °C in a humid environment, and were then subjected to tensile testing using a mechanical machine at the corresponding temperature conditions.

GNGP hydrogel containing methyl violet was cured on fresh pig skin to form an “N” shape, and was subjected to squeezing, pulling, and twisting to simulate mechanical interventions in practical applications. Furthermore, the GNGP hydrogel containing methyl violet was cured into a “Z” shape on fresh pigskin, immersed in simulated body fluids at 37 °C for 24 h, and then squeezed, pulled and twisted to simulate mechanical intervention in extreme fluid environments in applications. The GNGP hydrogel precursor solution (500 µL) was added dropwise onto microscope slides before rat heart, liver, spleen, lung, kidney, stomach, and intestinal tissues were placed overtop and cured using UV light. The slides were inverted to observe the adhesion. Injuries measuring 8 mm in length along the long axes were inflicted on rat small intestines, which were then sealed using the GNGP hydrogel and then injected with Agar Blue solution to observe leakage from the injury. Finally, a 6 × 8 mm oval defect was created in fresh porcine small intestine. After sealing the defect with GNGP hydrogel, the small intestine was placed in a 37 °C liquid environment. A peristaltic pump was used to circulate the simulated intestinal fluid containing methyl violet at a flow rate of 80 mL min^−1^. Leakage of the fluid was observed to evaluate the sealing effect of GNGP hydrogel.

Burst pressure testing was performed using human surgically resected small bowel tissue. After one end was sealed, the other end was connected to a syringe and an electronic manometer via a tee. A 4 mm puncture wound was created in the intestinal tube, which was then covered with fibrin glue and GNGP hydrogel (10 mm diameter). The syringe was pushed at a rate of 1 mL s^−1^ and the maximum value on the manometer was recorded to assess the bursting pressure of the hydrogel. A portion of the small intestine was taken at the end of the test, onto which the GNGP hydrogel was cured. It was then lyophilized and observed via SEM.

### Hydrogel Degradation and Histocompatibility

200 µL of GNGP hydrogel was in simulated intestinal fluid (1 mL) at 37 °C. The simulated intestinal fluid was changed immersed every 8 h. At the specified time, the hydrogel was removed for lyophilization and weighed. Place 200 µL of GNGP hydrogel in a 24‐well plate and then immerse it in simulated body fluid (1 mL) or simulated intestinal fluid (1 mL) at 37 °C. At the specified time, 100 µL of simulated body fluid was withdrawn from the corresponding wells. A triple quadrupole linear ion trap mass spectrometer (QTRAP6500+; Sciex, Framingham, MA) was used to measure the Nipam monomer concentration to evaluate the release of Nipam monomers from the GNGP hydrogel.

Hydrogel samples (200 µL) were fluorescently labeled by placing them in a solution containing Cy5.5‐NHS activated ester and incubating them overnight at room temperature in the dark. They were then washed three times in PBS in the dark to remove unreacted labeling reagents. The washed hydrogel samples were then implanted subcutaneously into the backs of SD rats to evaluate the biocompatibility and degradation of the material. The hydrogel's degradation was monitored continuously using a small animal imaging system (IVIS Lumina XRMS Series III; Perkin Elmer, MA) immediately after the surgery, on the third day following the surgery, and from the first to the eighth week. The rats were sacrificed at the second, fourth, and eighth weeks to obtain hydrogel‐embedded local tissues for H&E staining and immunofluorescence staining. Major organs such as hearts, livers, spleens, lungs, and kidneys were excised from rats at the eighth week to evaluate the possible toxic effects of the hydrogel on these vital organs. Meanwhile, 200 µL of hydrogel was implanted subcutaneously on the back of SD rats. The rats were sacrificed on the third day and from the first to the fourth week after the surgery. The hydrogels were separated, lyophilized, and weighed to evaluate the actual degradation of the hydrogels.

### Hydrogel Hemocompatibility

Sterilized GNGP hydrogel PBS extract, GNGP hydrogel precursor solution, PBS, and deionized water were added to 10% rat erythrocyte suspensions in a 1:1 ratio. After mixing, this was placed in a water bath at 37 °C and incubated for 3 h. The supernatant was collected following centrifugation at 3000 rpm for 10 min. The spectrophotometric optical density (OD) of the supernatant at 570 nm was determined using an enzymatic marker. Deionized water and PBS control absorbance values were recorded at OD_100_ and OD_0_, respectively. The hemolysis rate (HR) was calculated using the formula:

(3) $$\mathrm{HR}=\frac{\mathrm{OD}-OD_{0}}{OD_{100}-OD_{0}}\times 100\%$$

### Cell Culture

L929 and IEC‐6 cells were purchased from a commercial source (KeyGEN BioTech, Nanjing, China), and cultured in RPMI 1640 medium and high glucose Dulbecco's modified eagle medium (DMEM; Gibco, NY, USA), respectively. The medium contained 10% fetal bovine serum (Gibco) and 1% penicillin/streptomycin (Gibco). The cells were incubated at 37 °C in a humidified atmosphere of 5% CO_2_ and 95% air. The effect of the GNGP hydrogel on L929 fibroblasts was evaluated using live/dead cell staining and CCK8 kits. The apoptosis levels of L929 and IEC‐6 after 24 h of co‐culture with GNGP hydrogels were evaluated using the one‐step TUNEL Apoptosis Assay Kit. In vitro stretching experiments were conducted using a customized stretching device. IEC‐6 cells were seeded onto polydimethylsiloxane cast stretching chambers (Xuanyuan Technology Co., Ltd., Hangzhou, China) and incubated for 24 h, after which 15% strain stretching was performed. The effect of shear stress on the proliferation of IEC‐6 cells was assessed using an EdU staining kit (Servicebio Technology Co., Ltd., Wuhan, China). To inhibit the activity of YAP, vitexoporfin (1 × 10^−6^ m) was added to the culture medium 24 h before the stretching experiment.

### Antibacterial Properties

*E. coli* (ATCC 25922), *S. aureus* (ATCC 29213), and hyper‐virulent *K. pneumoniae* (NTUH‐K2044) were co‐cultured with different components of the hydrogel and examined for growth rate, colony forming density, live/dead cell ratio, and changes in bacterial morphology. Briefly, 1 × 10^8^ bacteria were inoculated in 5 mL of lysogeny broth (LB) medium and co‐cultured with 0.5 cm^3^ hydrogel samples that had undergone 1 h of UV irradiation. The cultures were incubated in a shaker at 37 °C for 24 h with shaking at 120 rpm. The medium (100 µL) was taken at specific times, and the absorbance at 600 nm was measured using an enzymatic marker on a Multiskan SkyHigh reader (Thermo‐Fisher Scientific) to quantify the bacterial growth rates. After 20 h of co‐cultivation, medium samples (100 µL) were collected from each group and the bacterial suspension was inoculated onto LB agar plates after being diluted 100‐fold with saline. The plates were placed in an incubator at 37 °C in a humidified atmosphere of 5% CO_2_ and 95% air for 24 h to observe bacterial colony formation. After 8 h of co‐culturing with the hydrogel, the bacteria were stained using propidium iodide and SYTO dyes, according to the manufacturer's instructions. The bacteria were then inoculated onto a bacterial counting plate and imaged using a confocal microscope. Bacterial suspension samples (1 mL) were collected after 20 h of co‐culturing and centrifuged at 4000 rpm for 10 min at 4 °C. The supernatant was discarded and, after the bacterial cells were fixed in place, their morphological changes were observed via SEM. Finally, the three bacterial suspensions at a standardized concentration of 0.5 McNeil units were homogeneously inoculated onto LB agar plates, and the different fractions of hydrogels (200 µL) that had been irradiated with UV light for 1 h were placed onto the surfaces of the plates. The plates were then placed in an incubator for 24 h. The effects of the hydrogel on bacterial growth were then observed and recorded.

### Animals

The male New Zealand white rabbits (12 weeks old) and male Sprague‐Dawley rats (8 weeks old) used in this study were anesthetized using isoflurane. The animals were housed under natural light‐dark cycle conditions at 25 °C with free access to food and water. All animal care and experimental procedures were performed in accordance with the Regulations for the Administration of Affairs Concerning Experimental Animals approved by the State Council of the People's Republic of China, and approved by the Institutional Animal Care and Use Committee of Jinling Hospital (Approval No. 2022DZGKJDWLS‐0074).

### Rabbit Intestinal Fistula Model Constructed by Stent Embedment

The effectiveness of the GNGP hydrogel in terms of promoting intestinal fistula healing was verified in a rabbit model of intestinal fistula. New Zealand white rabbits were fasted for 1 d. Trimmed 24 F T‐tubes were placed in the ileums of the rabbits, 10 cm from the end of the accompanying appendix. These T‐tubes were fixed to the intestinal wall using absorbable sutures. Each T‐tube was threaded out of the abdominal cavity from the lateral side of the abdominal wall, pulled tightly, and fixed to the abdominal wall with silk sutures. The external port of the T‐tube was blocked with gauze to prevent loss of intestinal fluid. Intraoperatively, cefoperazone sulbactam sodium solution (6 mL, 5 wt%) was administered to prevent infection, carprofen (4 mg kg^−1^) was given for pain relief, and cefoperazone sulbactam sodium (0.5 g) was added to the drinking water with postoperative fasting. Normal diets were gradually reintroduced beginning on day 4. The T‐tubes were removed on day 10 to form an animal model of intestinal fistula. Thirty‐two intestinal fistula rabbits were randomly divided into four groups of eight rabbits each. The control group did not undergo any treatment, while the fibrin glue, GGP hydrogel, and GNGP hydrogel groups were administered the respective treatments. The hydrogel was placed in an ice box before treatment and cured using UV light during injection. The animals were then returned to their cage. Half of the experimental animals were executed on days 3 and 7, and their fistulas and surrounding tissues were collected for subsequent analysis.

### Human Intestinal Fistula Samples

Intestinal fistulas and surgically resected normal intestinal tissues were obtained from patients who underwent intestinal fistula resection and digestive tract reconstruction surgery at the General Surgery Department of Jinling Hospital. Tissues were collected from three patients with intestinal fistula: two males with a mean age of 54.5 years and one female 57 years of age. All of the subjects gave written informed consent for this experiment. This study was approved by the medical ethics committee of Jinling Hospital (Approval No. 2023DZKY‐072‐01).

### Histological Assessment

Paraffin‐embedded sections were prepared, and H&E and Masson staining were performed to observe the tissue characteristics around the intestinal fistula, fistula healing, and collagen deposition. The pathological scale is shown in Table S3 (Supporting Information).

### Immunofluorescence and Immunohistochemistry

Microscope slides were hydrated and incubated with primary antibodies overnight at 4 °C. PBS was washed three times to remove excess primary antibody before being incubated with the appropriate secondary antibody at room temperature for 1 h. The slides were then immersed in citric acid repair solution, boiled for 10 min, and incubated with the second primary antibody and the appropriate secondary antibody. The nuclei of the cells were marked by incubation with 4′,6‐diamidino‐2phenylindole (Servicebio, Wuhan, China) for 10 min. All of the slides were then covered with sealer and other slides to reduce fluorescence attenuation. For immunohistochemical staining, after primary antibody incubation, the sections were incubated with corresponding horseradish peroxidase‐labelled secondary antibodies for 50 min at room temperature. Diaminobenzidine was used as a chromogen. The nuclei were counterstained with hematoxylin. Quantification of the stained tissues was performed using ImageJ software (version 1.53c, National Institutes of Health, Bethesda, MD).

### Western Blotting

Total protein from cells or tissues was extracted using RIPA buffer (Beyotime Biotechnology, Shanghai, China) with protease inhibitor (Beyotime Biotechnology) and phosphatase inhibitors (MedChemexpress, Shanghai, China). Protein concentrations were determined using a BCA protein kit (KeyGEN BioTech). The proteins were separated using sodium dodecylsulfate‐polyacrylamide gel electrophoresis and transferred to polyvinylidene difluoride membranes. The membranes were then incubated with primary antibodies overnight at 4 °C. Positive signals were scanned using a Tanon‐4600 chemiluminescent imaging system (Tanon Technology, Shanghai, China). The OD values of the protein bands were quantified using ImageJ and normalized according to the expression of GAPDH. Bands were detected using ultra‐sensitive enhanced chemiluminescence (Vazyme, China). The presented data are the means of three replicates.

### RNA‐seq

Total mRNA samples were extracted from tissues or cells using Trizol reagent (Sigma‐Aldrich), with three samples per group. Following this, cDNA libraries were constructed using the VAHTS Universal V6 RNA‐seq Library Prep Kit for Illumina (NR604‐02; San Diego, CA) and sequenced on the Illumina NovaSeq 6000 platform. Clean reads filtered from the raw reads were mapped to the references using Hisat. DESeq was used to define DEGs as those with adjusted *P*‐values of <0.05 and absolute ‐fold changes of ≥2.0 or 1.5. Differential expression analysis was performed using the DESeqR software package. GO and KEGG databases were enriched using the clusterProfiler R software package for DEG analysis. The raw data files of all RNA‐seq analyses were deposited into the NCBI Sequence Read Archive database under accession numbers PRJNA1002855, PRJNA1055669, and PRJNA1086025.

### Finite Element Analysis

Finite element simulations of the process of intestinal fistula contraction by the GNGP hydrogels were conducted using ABAQUS software 2018 (Dassault Systemes Simulia Corp., Providence, RI). Briefly, a negative thermal expansion coefficient was used to simulate the thermal response behavior of the GNGP hydrogel. The tissue surrounding the intestinal fistulas and hydrogels were constructed as cylindrical models with diameters of 60 mm and thicknesses (i.e., heights) of 20 mm. The model consisted of intestinal (2 mm), muscle (15 mm), and skin (3 mm) samples with 8 mm diameter hydrogels set in their centers. The intestinal, muscle, and skin samples were modeled as Ogden hyperplastic materials and substituted into the models according to the parameters detailed in Table S4 (Supporting Information).^[^ ^37^ ^]^ The edges of the model were fixed and the temperature was to trigger the active response of the hydrogel.

### Statistical Analysis

The data were analyzed using GraphPad Prism (version 9.0), and all data are shown as the mean ± SEM. An unpaired Student's *t*‐test (two tailed) was used to compare the two groups. One‐way analysis of variance (ANOVA) or two‐way ANOVA (with two influencing factors) was used for multiple group comparisons. Tukey's post hoc tests were used as needed. A *p*‐value of less than 0.05 was considered statistically significant.

## Conflict of Interest

The authors declare no conflict of interest.

## Author Contributions

Conceptualization, R.J., L.J., H.J., and L.Z.; data curation, L.Z., H.J., L.J., and C.K.; formal analysis, L.Z., H.J., L.Y., and L.S.; investigation, L.Z., H.J., C.K., Q.G., and M.R.; methodology, L.Z., L.J., and H.J.; resources, R.J., W.P., W.X., and H.J.; validation, L.Z., H.J., and T.Y.; visualization, L.Z., L.J., and Y.S.; writing – original draft, L.Z., L.J., W.P., W.X., and R.J.; project administration, W.P., W.X., and R.J.; supervision, R.J., W.P., Z.Y., and W.X.; funding acquisition, R.J., H.J., and W.X. All authors have read the manuscript, agreed to its content, and approved its submission.

## Supporting information



Supporting Information

Supplemental Video 1

Supplemental Video 2

## Data Availability

The data that support the findings of this study are available from the corresponding author upon reasonable request.
