# Supplementary material for: Mechanically Active Hydrogel for Healing Intestinal Fistulas through the YAP‐Mediated Mechanosensitization of Intestinal Epithelial Cells
Source: Adv Sci (Weinh). 2025 Aug 11;12(43):e10717. doi: 10.1002/advs.202510717 (PMC12631856; doi:10.1002/advs.202510717)
Supplement: Supplementary file 1 — Supporting Information [file ADVS-12-e10717-s001.pdf]

Supporting Information

Mechanically active hydrogel for healing intestinal fistulas through the YAP-mediated mechanosensitization of intestinal epithelial cells

Ze Li[1,2], Jiayang Li[1], Kang Chen[3], Guiwen Qu[4], Shuanghong Yang[5], Sicheng Li[1], Ye Liu[4], Yitian Teng[1], Rui Ma[3], Jinjian Huang[1, 3, 4, 5, *], Peige Wang[2,*], Jianan Ren[1, 2, 3, 4, 5,*], Xiuwen Wu[1, 2, 5 ,*]

[1] Research Institute of General Surgery, Jinling Hospital, Affiliated Hospital of Medical School, Nanjing University, Nanjing 210002, P.R. China.

[2] Department of Emergency Surgery, The Affiliated Hospital of Qingdao University, Qingdao 266000, P.R. China.

[3] Research Institute of General Surgery, Jinling Hospital, Nanjing Medical University, Nanjing 210002, P.R. China.

[4] School of Medicine, Southeast University, Nanjing 211189, P.R. China.

[5] Jinling Clinical Medical College, Nanjing University of Chinese Medicine, Nanjing 210002, P.R. China.

[*] Correspondence to:

Jinjian Huang, Research Institute of General Surgery, Jinling Hospital, Affiliated Hospital of Medical School, Nanjing University, Nanjing 210002, P.R. China. E-mail: jinjian_huang@seu.edu.cn.

Peige Wang, Department of Emergency Surgery, The affiliated hospital of Qingdao University, Qingdao 266000, P.R. China. E-mail: wpgzyz@163.com.

Xiuwen Wu, Research Institute of General Surgery, Jinling Hospital, Affiliated Hospital of Medical School, Nanjing University, Nanjing 210002, P.R. China. E-mail: wuxiuwen@nju.edu.cn.

Jianan Ren, Research Institute of General Surgery, Jinling Hospital, Affiliated Hospital of Medical School, Nanjing University, Nanjing 210002, P.R. China. E-mail: Jiananr@nju.edu.cn, jiananr@gmail.com;

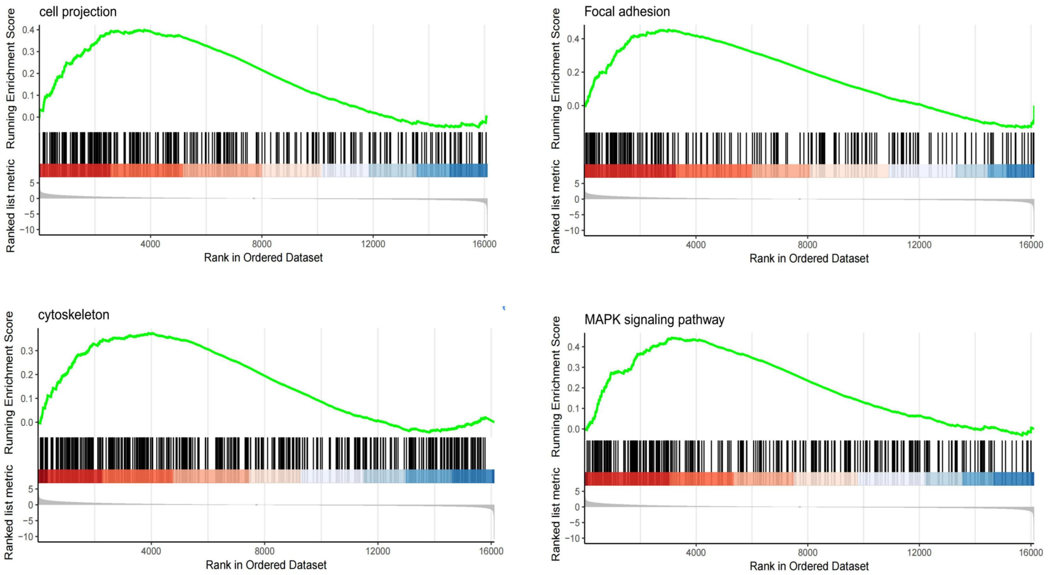

Fig. S1: Gene set enrichment analysis (GSEA) of differentially expressed genes in intestinal fistula and normal intestinal tissues.

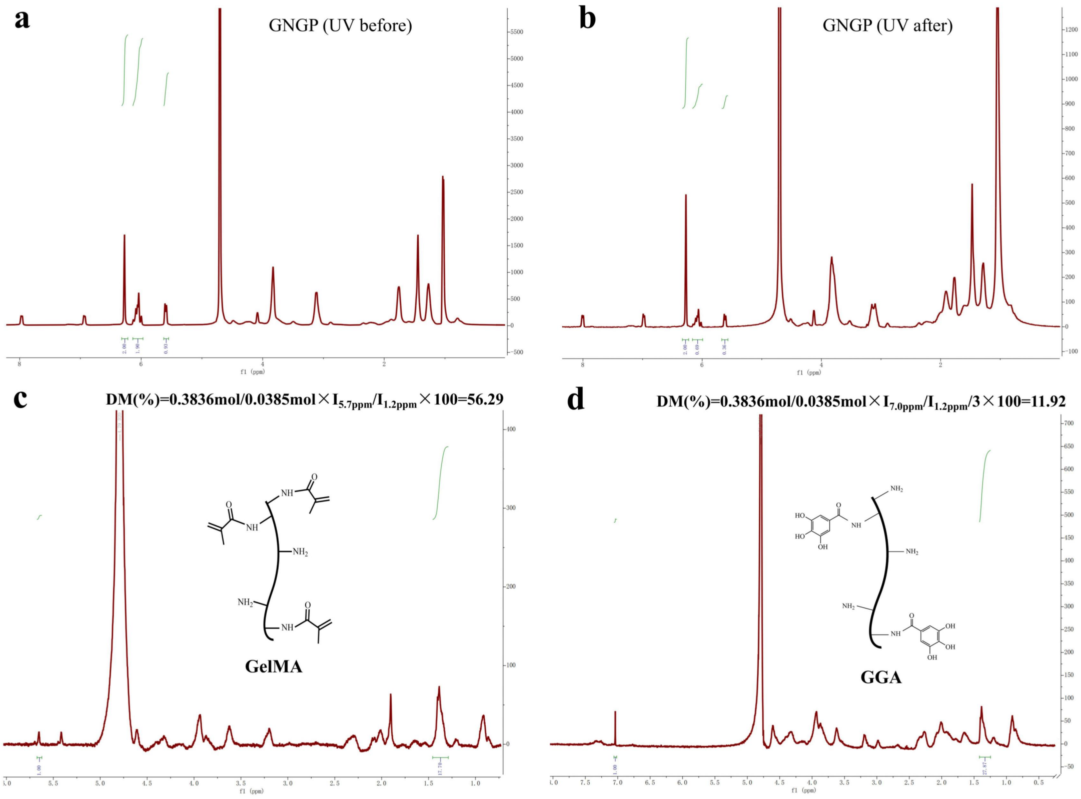

Fig. S2: NMR spectra of GelMA, GGA and GNGP. (a, b) Quantitative NMR spectra of GNGP hydrogel before and after UV irradiation. (c) NMR spectra of GelMA and formula for calculation of substitution degree. (d) NMR spectra of GGA and formula for calculation of substitution degree.

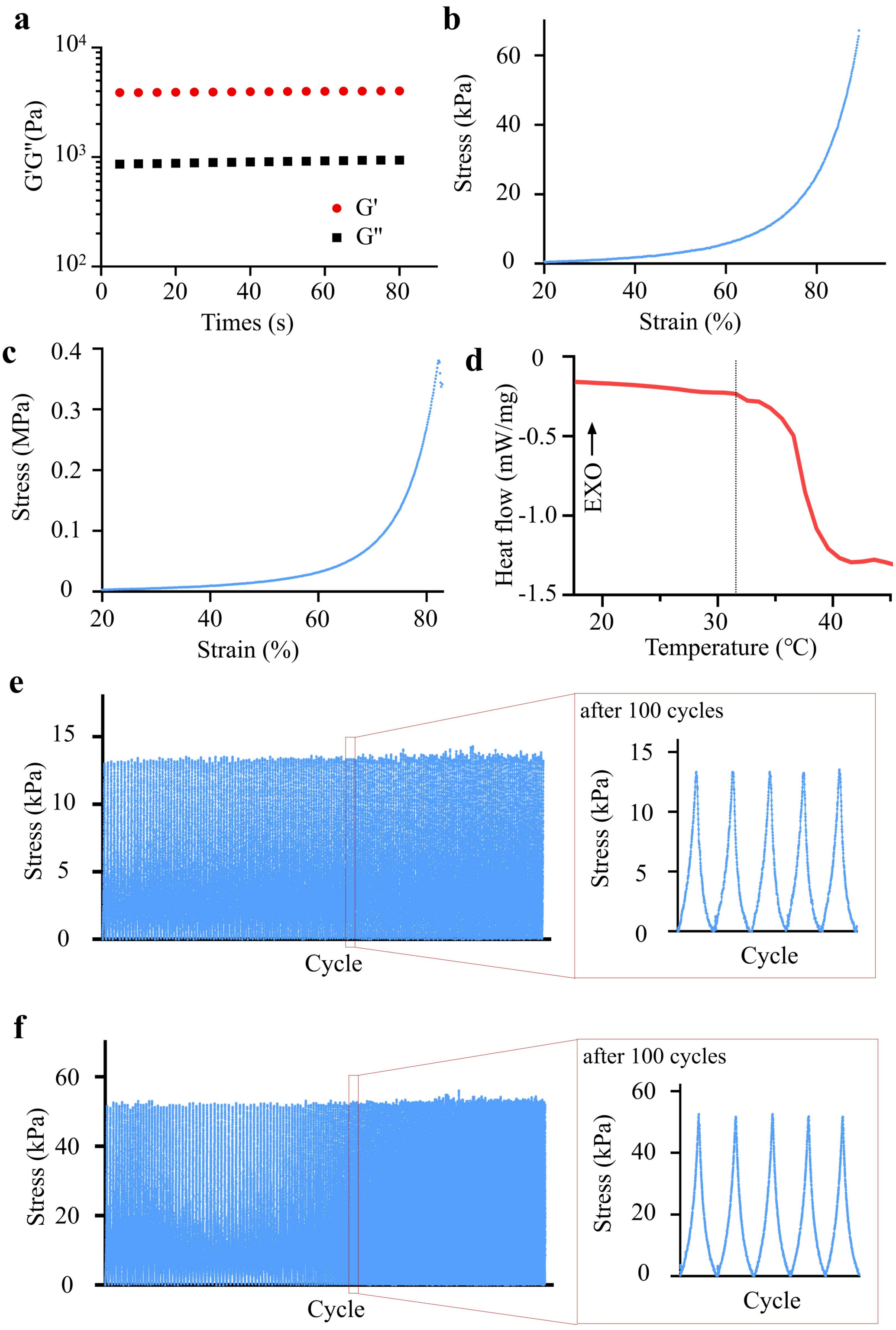

Fig. S3: Characteristics of mechanically active hydrogel (GNGP). (a) Rheological characterization of GNGP hydrogels at 37°C. (b) Compression test of GNGP hydrogel at 25°C. (c) Compression test of GNGP hydrogel at 37°C. (d) DSC curve of GNGP hydrogel. (e) Compression cycling test of GNGP hydrogel at 25°C for 200 cycles. (f) Compression cycling test of GNGP hydrogel at 37°C for 200 cycles.

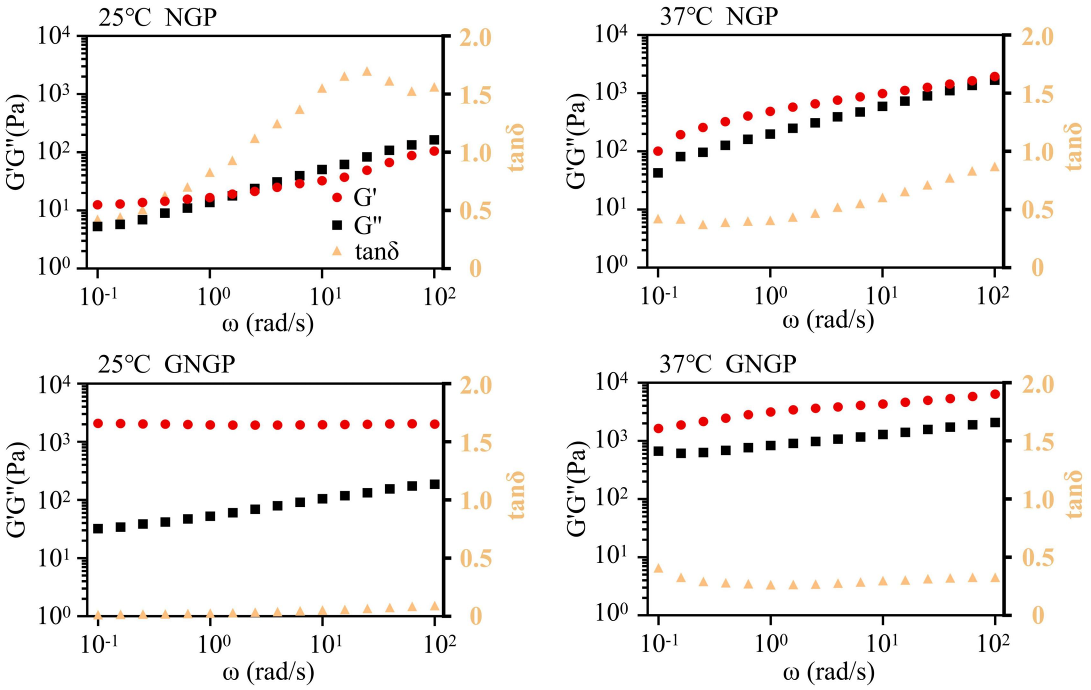

Fig. S4: Rheological characterization of GNGP and NGP hydrogels at 25 and 37 °C. After the introduction of GelMA, the energy storage modulus of the hydrogel was increased by several tens of times, while the tan δ was lowered.

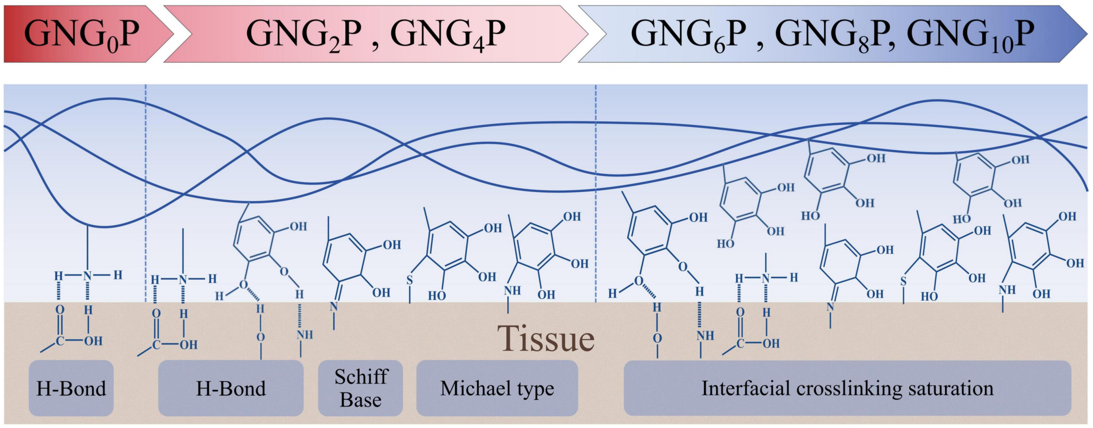

Fig. S5: Schematic representation of GNGP hydrogel-tissue interface adhesion.

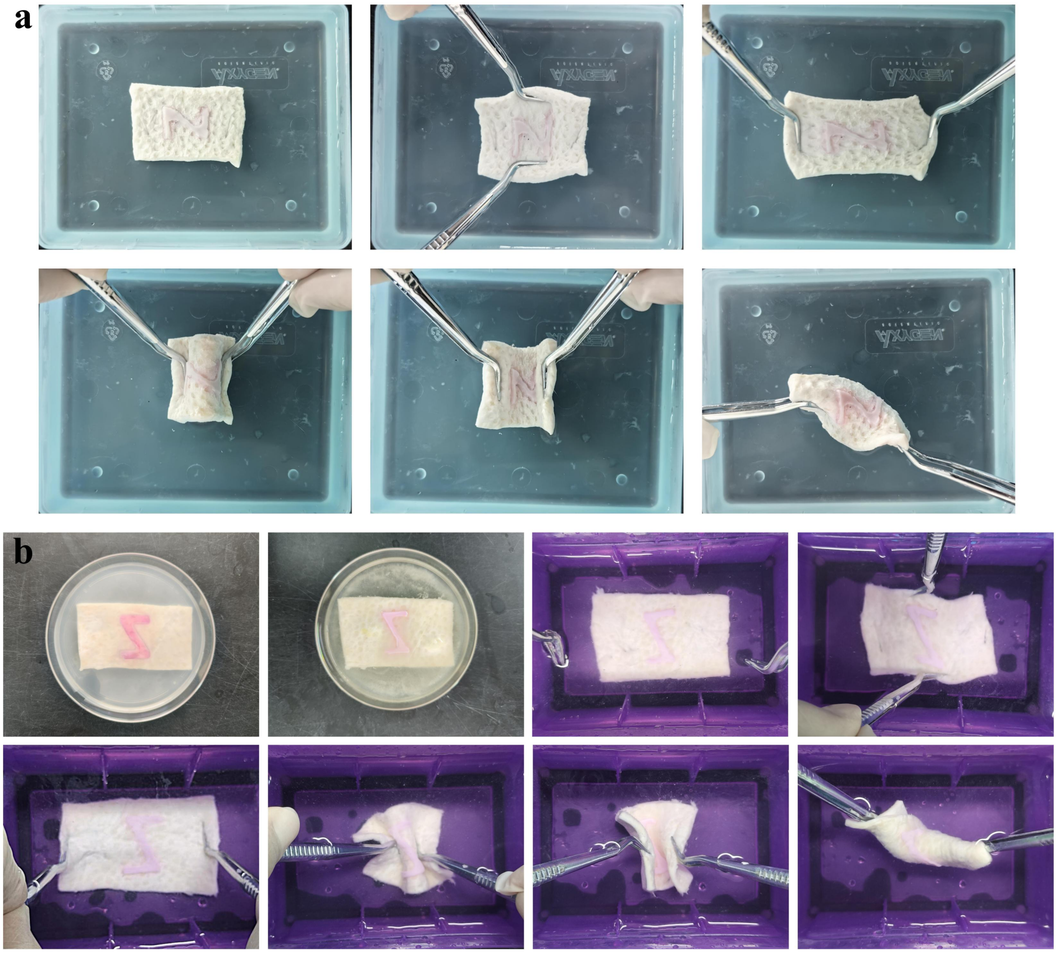

Fig. S6: Adhesion stability of mechanically active hydrogel (GNGP) in liquid environments. GNGP hydrogel forms a stable adhesion and resists multiple deformations immersion in simulated body fluids (a) and simulated intestinal fluids (b) at 37°C for 24 hours.

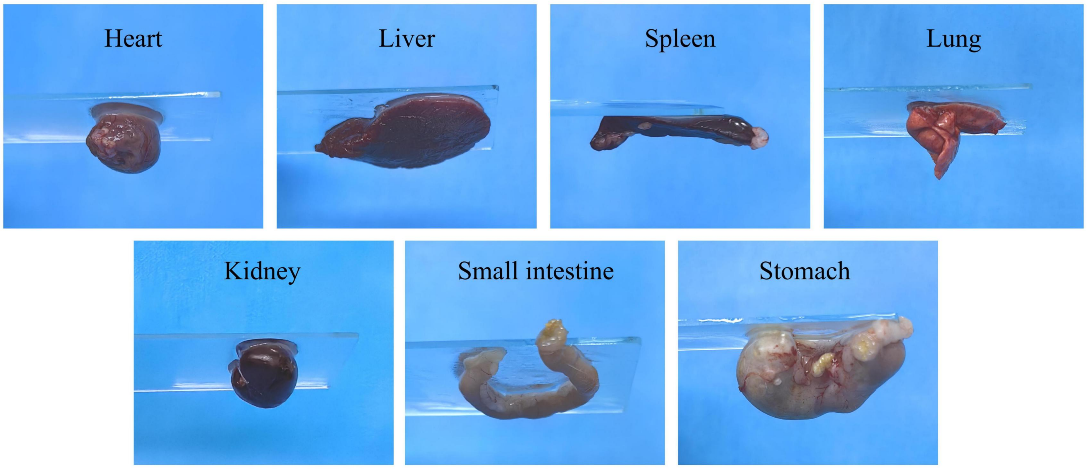

Fig. S7: Our mechanically active hydrogel (GNGP) showed good adhesion to heart, liver, spleen, lung, kidney, small intestine, and stomach tissues.

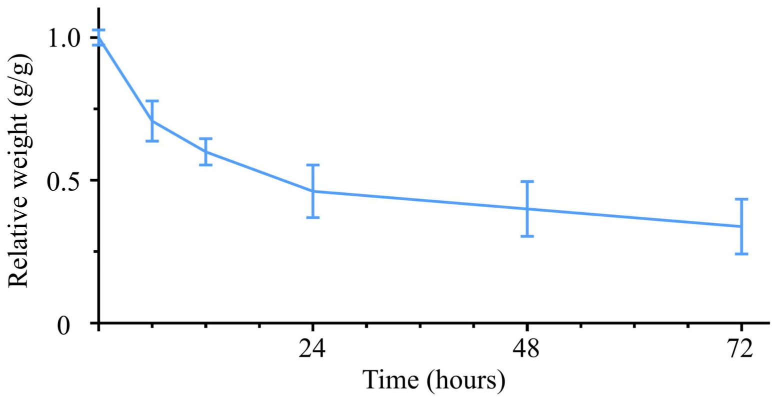

Fig. S8: Degradation profile of mechanically active hydrogel (GNGP) in simulated intestinal fluid at 37°C (n = 3).

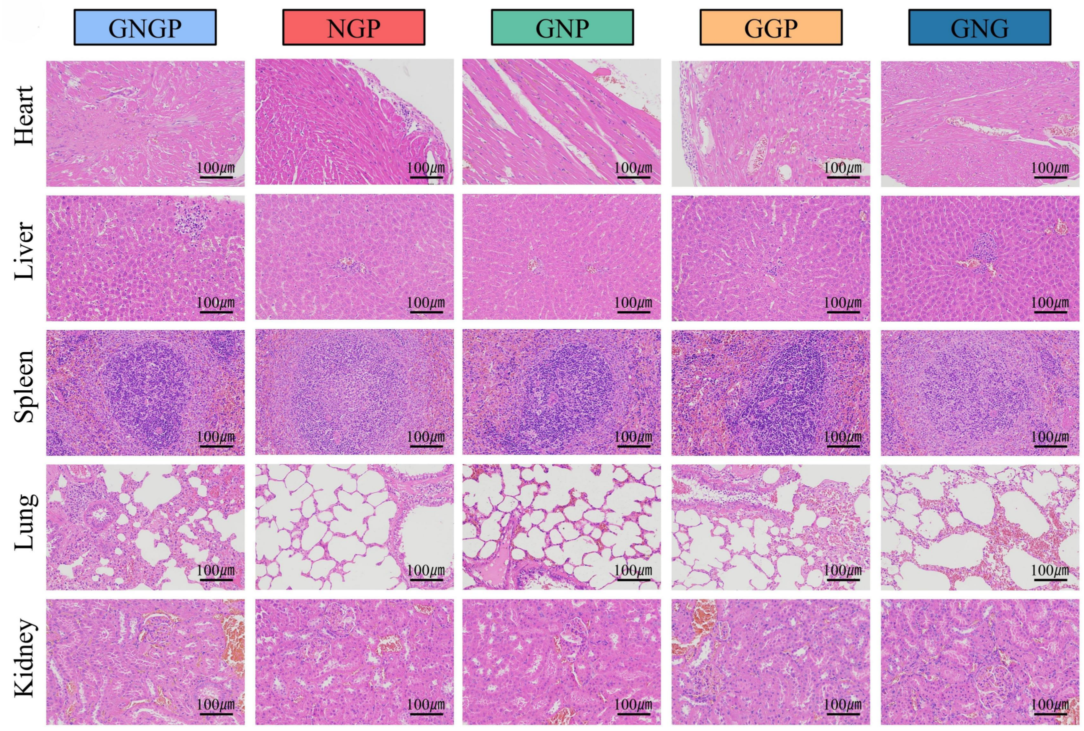

Fig. S9: The GNGP hydrogel showed no toxic effects on major organs *in vivo*. Representative pictures of HE staining of major organs after the hydrogel was embedded in rats for 8 weeks, showing no toxic effects or damage in any of the groups.

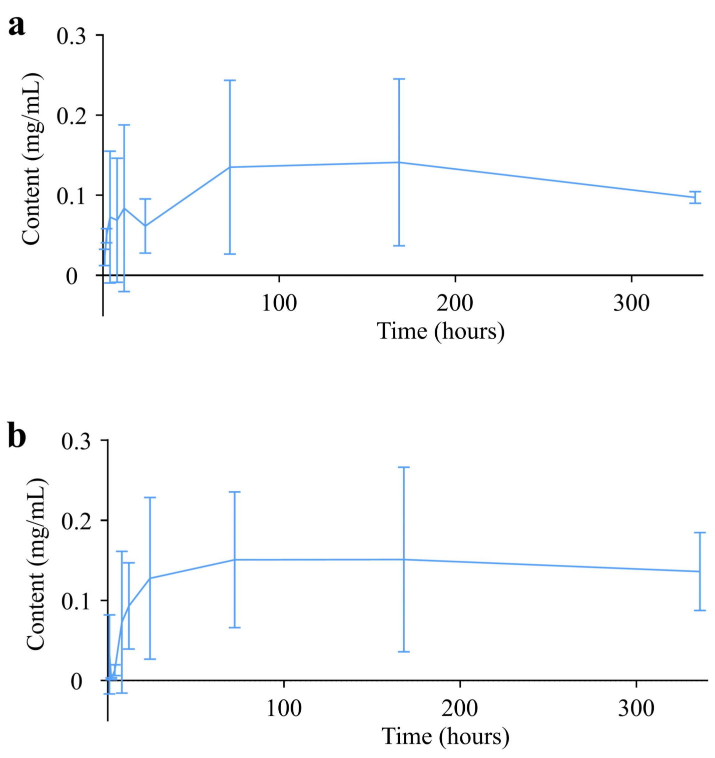

Fig. S10: The concentration of Nipam monomers decomposed by mechanically active hydrogel (GNGP) at 37°C (n = 3). (a) In simulated body fluids. (b) In simulated intestinal fluid.

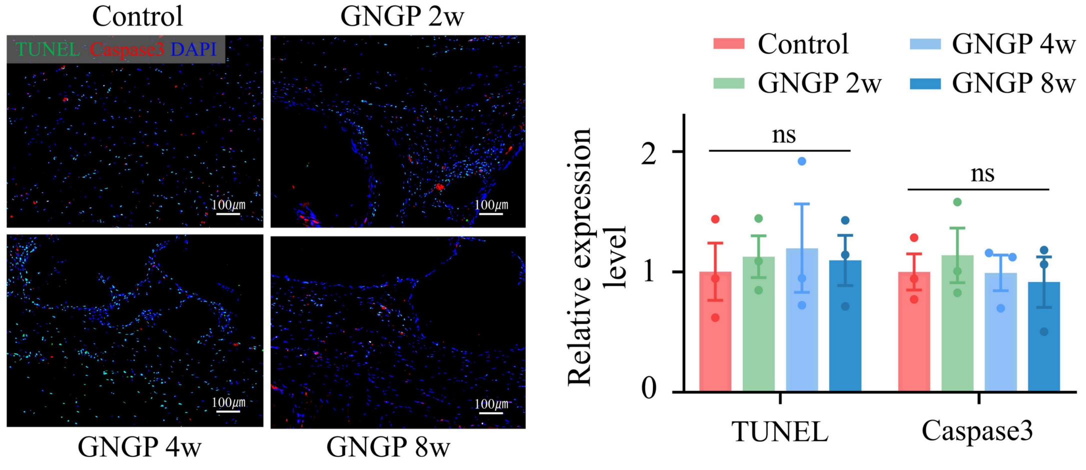

Fig. S11: Apoptosis detection of mechanically active hydrogel (GNGP) degraded in rats. GNGP hydrogel didn't lead to apoptosis of surrounding cells after subcutaneous degradation in rats (n = 3). The *p* values in the figure are determined by one-way ANOVA followed by Tukey's multiple comparisons test. Data are presented as mean ± SD. ns, not significant.

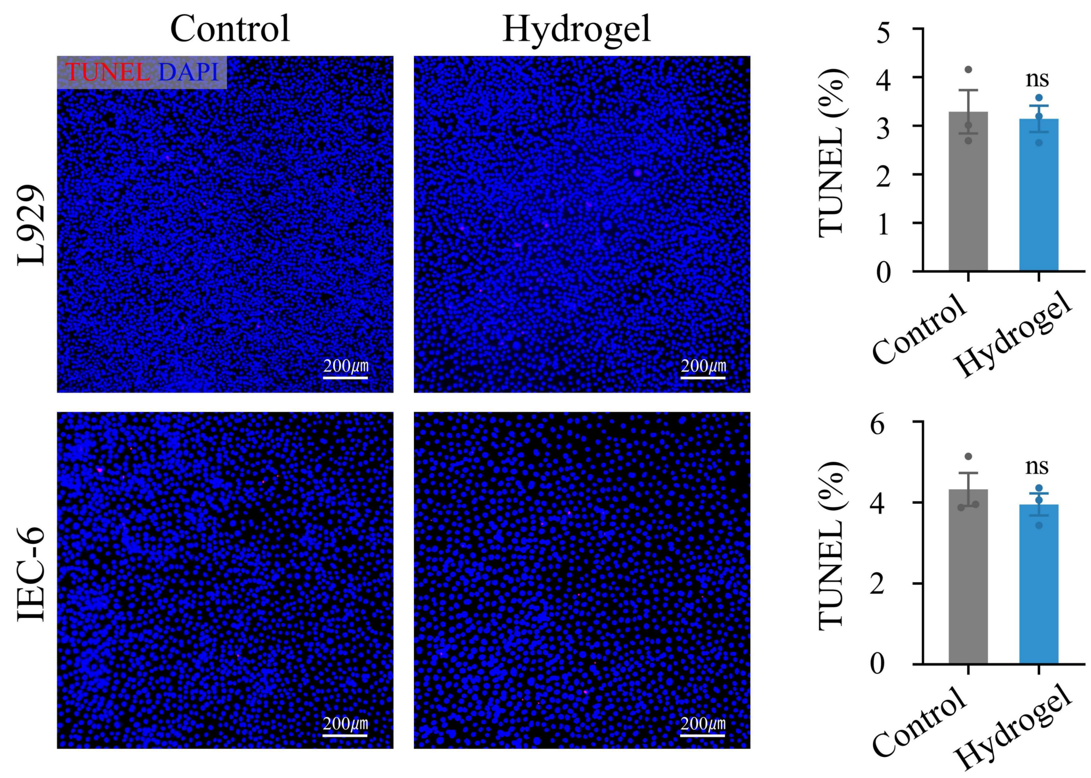

Fig. S12: Apoptosis after co-culture of L929 and IEC-6 with mechanically active hydrogel (n = 3). The *p* values in the figure are determined by two-sided unpaired *t* test. Data are presented as mean ± SD. ns, not significant.

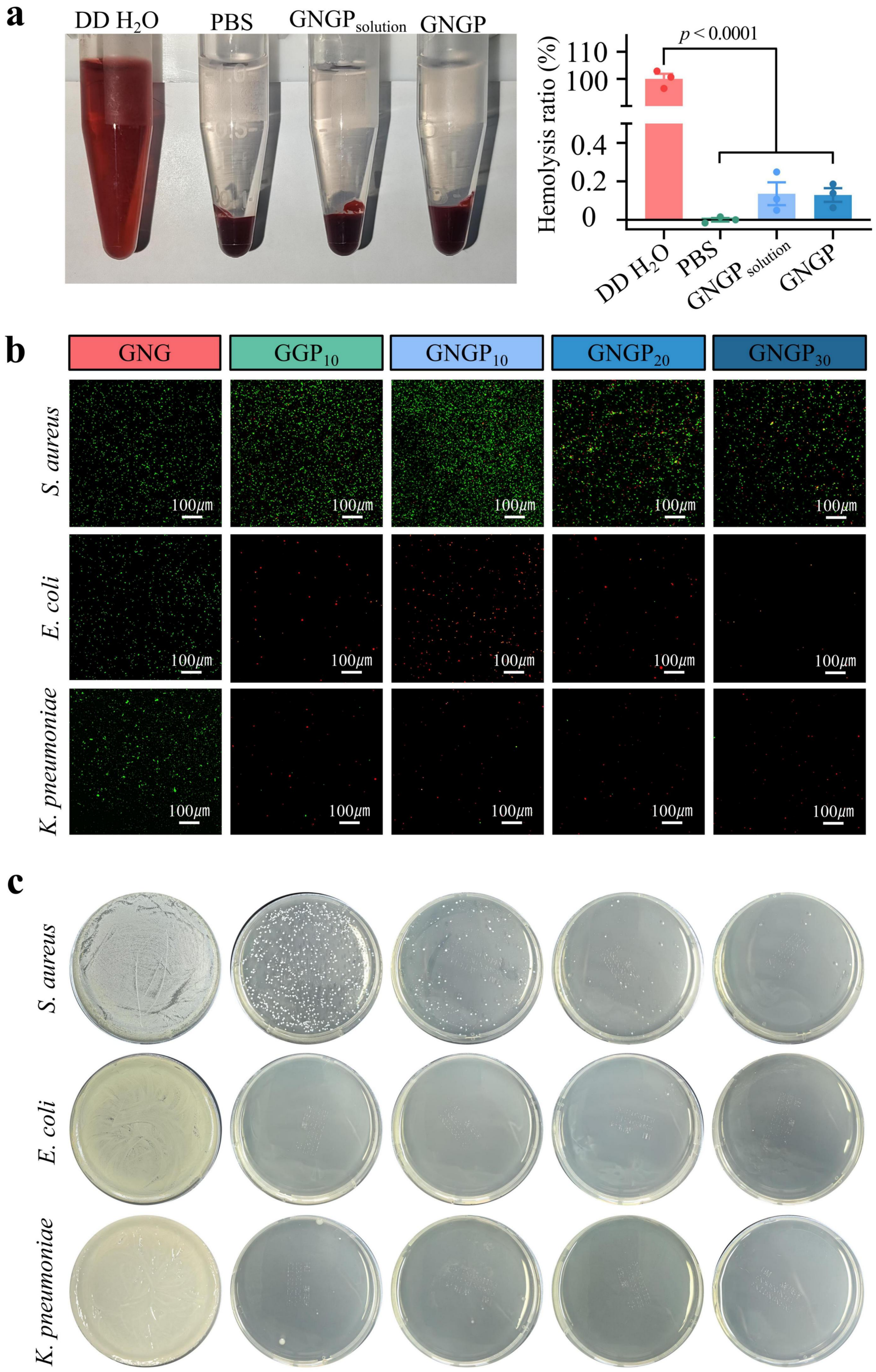

Fig. S13: The GNGP hydrogel showed excellent antimicrobial properties and hemocompatibility. (a) Hemolytic assay of the hydrogel with its precursor solution, showing excellent hemocompatibility with its precursor solution (n = 3). (b) Live/dead staining of *Staphylococcus aureus*, *Escherichia coli*, and highly-virulent *Klebsiella pneumoniae* after 8 hours of co-culturing with different hydrogel components. (c) Bacterial counts of different components of GNGP hydrogels after co-culturing with pathogenic bacteria were determined using the plate counting method. The p values in the figureare determined by one-way ANOVA followed by Tukey's multiple comparisons test. Data are presented as mean ± SD.

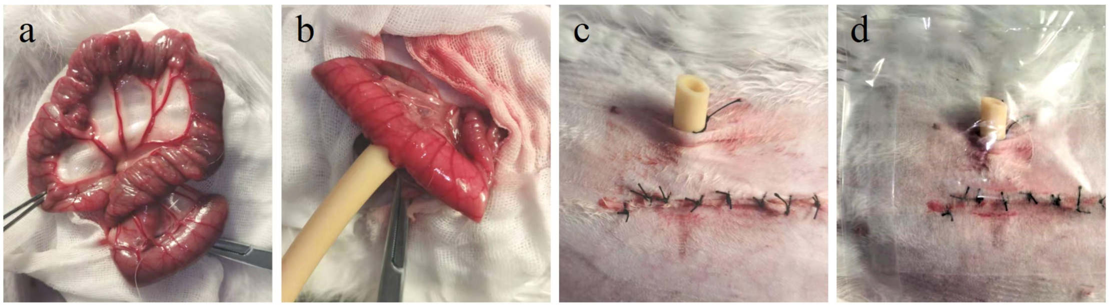

Fig. S14: Procedure for constructing a rabbit animal model of intestinal fistula. (a) After opening the abdomen, the ileum was found to be located 10 cm from the companion position with the end of the appendix (as indicated by the forceps). (b) The T-tube was left in place after incising the intestine. (c) The T-tube was threaded out of the abdominal wall and secured. (d) The T-tube was sterilized, and a stoma bag was affixed.

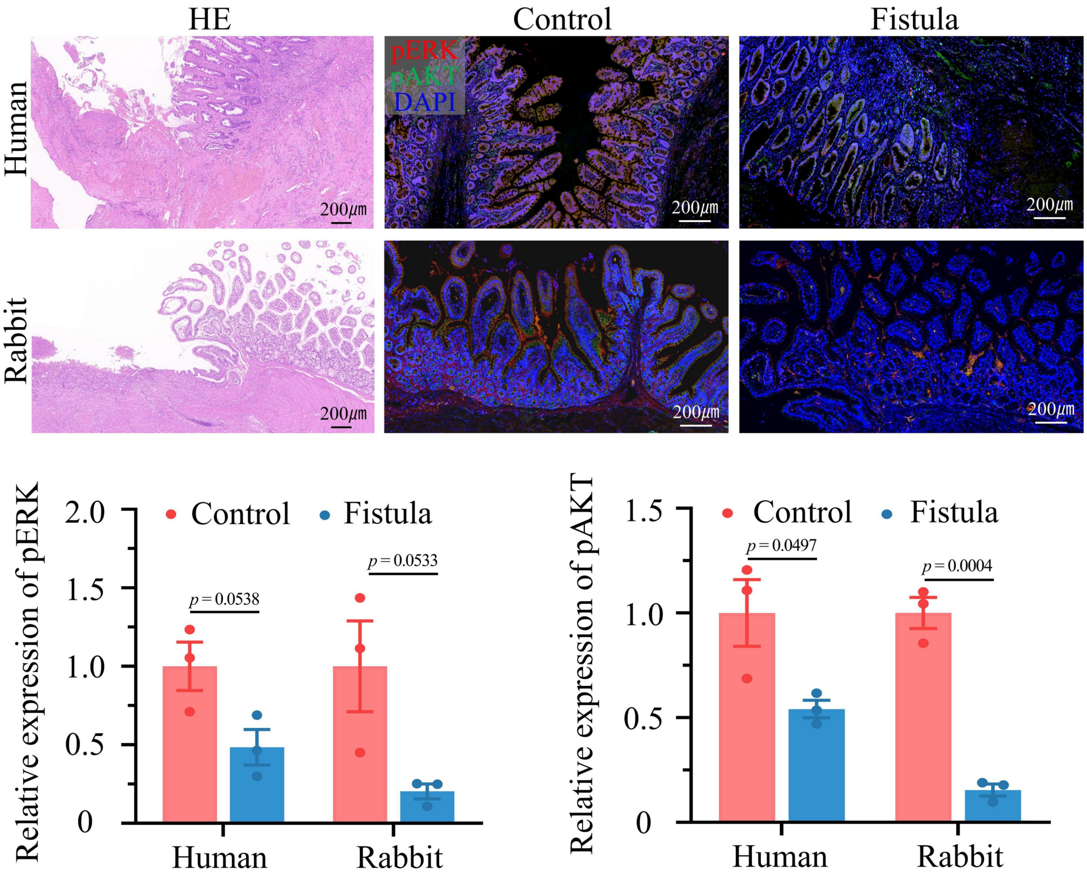

Fig. S15: Histological characterization of rabbit and human intestinal fistulas. Phosphorylation of ERK and AKT (key factors of the MAPK and PI3K signaling pathways, respectively) was inhibited in both human and rabbit intestinal fistulas (n = 3). The p values in the figure are determined by two-sided unpaired t test. Data are presented as mean ± SD.

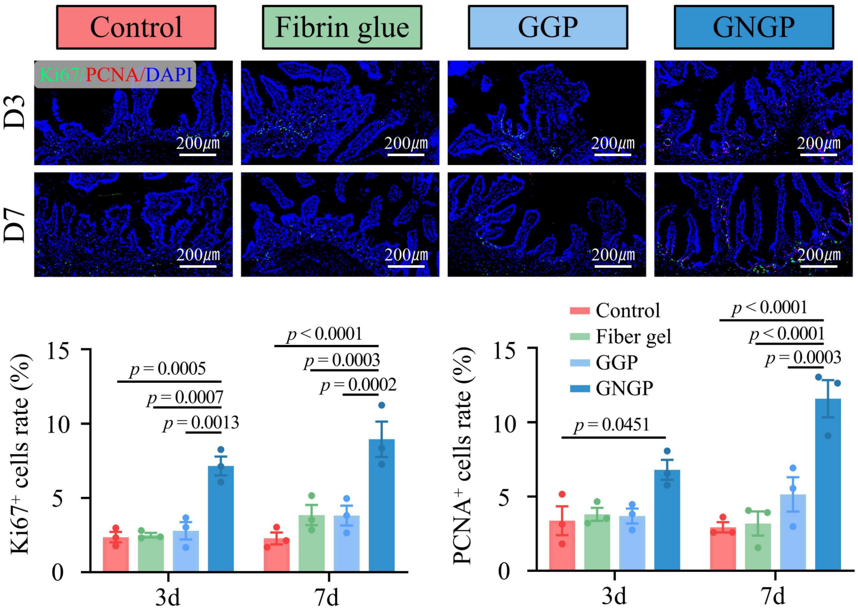

Fig. S16: GNGP hydrogel sealing treatment promotes regeneration of intestinal mucosa in rabbit intestinal fistula. Representative immunofluorescence images and quantitative analysis of Ki67 and PCNA (n = 3). The *p* values in the figure are determined by two-way ANOVA followed by Tukey's multiple comparisons test.

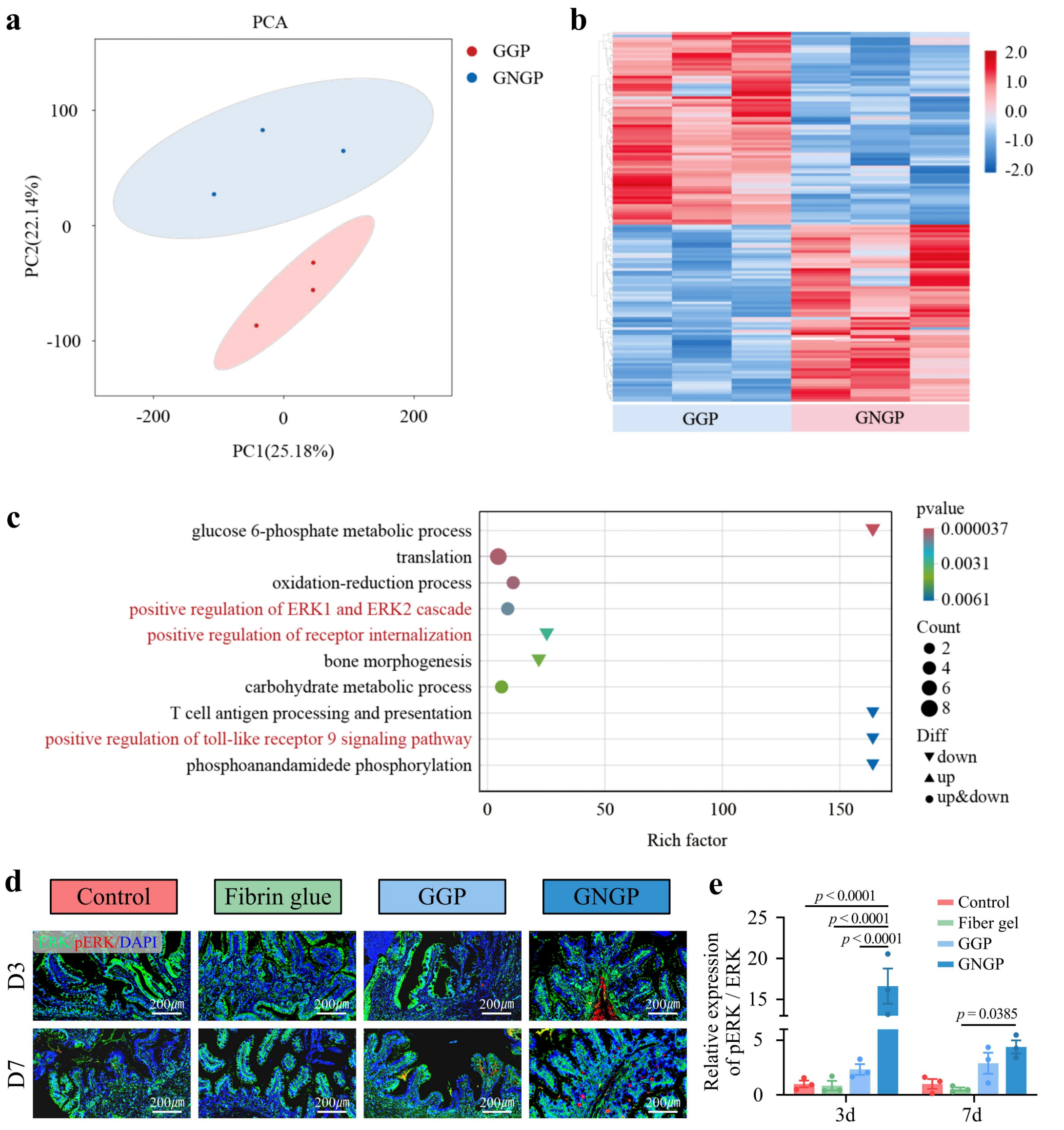

Fig. S17: The MAPK signaling pathway was activated after rabbit intestinal fistulas were treated with GNGP hydrogel. (a) Principal component analysis of the intestinal fistula transcriptome after 7 days of treatment with our mechanically active GNGP hydrogel and a GGP hydrogel. (b) Heat map of differentially expressed genes. (c) GO bioprocess analysis of differentially expressed genes. (d) Immunofluorescent staining of an intestinal fistula with (e) quantitative analysis (n = 3). The MAPK signaling pathway was activated in the intestinal fistulas of the GNGP hydrogel group. The p values in the figure (e) are determined by two-way ANOVA followed by Tukey's multiple comparisons test. Data are presented as mean ± SD.

Table S1. Quantitative NMR analysis of GNGP hydrogels before and after UV irradiation.

Sample	Weight (mg)	Internal standard (Maleic acid, mg)	$I_{6.0ppm}$	$I_{6.3ppm}$	Molecular weight of maleic acid	Concentration of olefins (mol/g)	Double-bond conversion
GNGP (UV before)	70.1	12	1.90	2.00	116.07	0.00280	80%
GNGP (UV after)	111	10.4	0.69	2.00	116.07	0.00056	

Table S2. Concentrations of components of hydrogels with different abbreviations.

Abbreviation	GelMA(w/v%)	NPIAM (w/v%)	GGA (w/v%)	ε-PL (w/v%)
GNGP	5	25	4	20
GNG_0P	5	25	-	20
GNG_2P	5	25	2	20
GNG_4P	5	25	4	20
GNG_6P	5	25	6	20
GNG_8P	5	25	8	20
$GNG_{10}P$	5	25	10	20
$GN_{20}GP$	5	20	4	20
$GN_{25}GP$	5	25	4	20
$GN_{30}GP$	5	30	4	20
NGP	-	25	4	20
GNP	5	25	-	20
GGP	5	-	4	20
GNG	5	25	4	-
GGP_{10}	5	-	4	10
$GNGP_{10}$	5	25	4	10
$GNGP_{20}$	5	25	4	20
$GNGP_{30}$	5	25	4	30

Table S3. Histopathological scale for intestinal fistula healing

Pathological characteristics	Description	Scale	Score
Re-epithelialization<br>% = [Distance of epithelial cover on both sides] / [Distance of initial defect] ×100	Complete<br>Partial<br>Missing	95-100<br>< 95, >0<br>0	2<br>1<br>0
Intestinal epithelial thickness index (ETI)<br>ETI = [Average thickness of wound epithelium] / [Average thickness of normal intestinal epithelium]×100	Complete<br>Partial<br>Missing	≥80<br>< 80, >0<br>0	2<br>1<br>0
Morphology of intestinal epithelial cells<br>% = [Number of epithelial cells with morphological abnormalities such as swelling and crumpling and necrosis, heterogeneous hyperplasia, etc.] / [Total number of epithelial cells in healing tissue] × 100	Normal<br>Partially abnormal<br>Abnormal	< 5<br>< 15, ≥5<br>≥15	2<br>1<br>0
Muscle layer healing index<br>%=[Distance of initial defect - distance of muscle layers on both sides] / [Distance of initial defect]×100	Complete<br>Partial<br>Missing	95-100<br>< 95, >0<br>0	2<br>1<br>0

Table S4. Ogden coefficients for component properties in finite element simulation.

Materials	α	μ (kPa)
Intestine	5.97	5.05
Muscle	8.97	2.24
Skin	33	4.9
